# NAD+ Biosynthesis Ameliorates a Zebrafish Model of Muscular Dystrophy

**DOI:** 10.1371/journal.pbio.1001409

**Published:** 2012-10-23

**Authors:** Michelle F. Goody, Meghan W. Kelly, Christine J. Reynolds, Andre Khalil, Bryan D. Crawford, Clarissa A. Henry

**Affiliations:** 1 Graduate School of Biomedical Sciences, University of Maine, Orono, Maine, United States of America; 2 Department of Biology, Colby College, Waterville, Maine, United States of America; 3 Institute for Molecular Biophysics, The Jackson Laboratory, Bar Harbor, Maine, United States of America; 4 Department of Mathematics and Statistics, University of Maine, Orono, Maine, United States of America; 5 University of New Brunswick, Fredericton, New Brunswick, Canada; 6 School of Biology and Ecology, University of Maine, Orono, Maine, United States of America; Howard Hughes Medical Institute, Program in Genomics, Children's Hospital, Boston, MA, United States of America

## Abstract

Muscular dystrophies are common, currently incurable diseases. A subset of dystrophies result from genetic disruptions in complexes that attach muscle fibers to their surrounding extracellular matrix microenvironment. Cell-matrix adhesions are exquisite sensors of physiological conditions and mediate responses that allow cells to adapt to changing conditions. Thus, one approach towards finding targets for future therapeutic applications is to identify cell adhesion pathways that mediate these dynamic, adaptive responses in vivo. We find that nicotinamide riboside kinase 2b-mediated NAD+ biosynthesis, which functions as a small molecule agonist of muscle fiber-extracellular matrix adhesion, corrects dystrophic phenotypes in zebrafish lacking either a primary component of the dystrophin-glycoprotein complex or integrin alpha7. Exogenous NAD+ or a vitamin precursor to NAD+ reduces muscle fiber degeneration and results in significantly faster escape responses in dystrophic embryos. Overexpression of paxillin, a cell adhesion protein downstream of NAD+ in this novel cell adhesion pathway, reduces muscle degeneration in zebrafish with intact integrin receptors but does not improve motility. Activation of this pathway significantly increases organization of laminin, a major component of the extracellular matrix basement membrane. Our results indicate that the primary protective effects of NAD+ result from changes to the basement membrane, as a wild-type basement membrane is sufficient to increase resilience of dystrophic muscle fibers to damage. The surprising result that NAD+ supplementation ameliorates dystrophy in dystrophin-glycoprotein complex– or integrin alpha7–deficient zebrafish suggests the existence of an additional laminin receptor complex that anchors muscle fibers to the basement membrane. We find that integrin alpha6 participates in this pathway, but either integrin alpha7 or the dystrophin-glycoprotein complex is required in conjunction with integrin alpha6 to reduce muscle degeneration. Taken together, these results define a novel cell adhesion pathway that may have future therapeutic relevance for a broad spectrum of muscular dystrophies.

## Abstract 2

NAD+ improves muscle tissue structure and function in dystrophic zebrafish by increasing basement membrane organization.

## Author Summary

A variety of diseases, both inherited and acquired, affect muscle tissues in humans. Critical to muscle homeostasis is the anchoring of muscle fibers to their surrounding microenvironment through cell adhesion complexes that help to resist the repeated stress experienced during muscle contraction. Genetic mutations in these complexes weaken this mechanical attachment, making fibers more susceptible to damage and death. The resulting increased fiber degeneration can eventually lead to progressive muscle-wasting diseases, known collectively as muscular dystrophies. Although clinical trials are ongoing, there is presently no way to cure the loss of muscle structure and function associated with these diseases. We identified a novel cell adhesion pathway involving integrin alpha6 that promotes adhesion of muscle cells to their microenvironment. Here, we show that activation of this pathway not only significantly reduces muscle degeneration but also improves the swimming ability of dystrophic zebrafish. We explore the likely benefits and limitations of this pathway in treating symptoms of congenital muscular dystrophies. Our findings suggest that activation of this pathway (for example, by boosting levels of NAD+) has the potential to ameliorate loss of muscle structure and function in multiple muscular dystrophies.

## Introduction

The extracellular matrix (ECM) connects to the intracellular actin cytoskeleton via transmembrane receptor complexes. These adhesion complexes not only serve as scaffolds for tissue architecture but also function as sensors of physiological change and signal transduction hubs. Thus, adhesion complexes facilitate cell adaptation to changing conditions during development, aging, injury response, and disease. Perhaps not surprisingly, the onset and progression of many diseases is affected by disrupted cell-ECM interactions. Despite the fact that modulating cell-ECM adhesion could be exploited for therapeutic purposes, the dynamic regulation of cell-ECM adhesion in vivo is not well understood.

Muscles and tendons function as an integrated unit to transduce force to the skeletal system and stabilize joints. Cell-ECM adhesions mechanically link muscles to tendons and are required for muscle physiology and homeostasis. Many muscle diseases, such as Duchenne, Becker, Merosin-deficient muscular dystrophies, and congenital muscular dystrophy (CMD) with integrin deficiency, result from mutations that disrupt adhesion of muscle fibers to their surrounding basement membrane (BM), a substructure within the ECM. This weakened link between muscle fibers and the BM results in increased susceptibility to fiber damage and death during repeated cycles of contraction and relaxation. Due to the continuous bidirectional communication between cells and their surrounding BM, muscle atrophy is also accompanied by degeneration of the ECM microenvironment [1]. As the BM also provides support for satellite cells that mediate muscle repair, augmenting the BM is a potential strategy to improve muscle structure and regenerative capacity [2]–[4].

Muscle fibers are known to utilize two receptor complexes to adhere to laminin in the BM, the dystrophin-glycoprotein complex (DGC) and integrin alpha7beta1 heterodimers. Disruption of components in either complex can lead to muscle disease. Dystroglycan (UniProtKB accession number Q8JHU7_DANRE) is a major component of the DGC and consists of two subunits (alpha and beta) transcribed from the same locus, DAG1 [5]. Beta-Dystroglycan is a transmembrane subunit that indirectly links to the intracellular cytoskeleton, and alpha-Dystroglycan binds to laminin and agrin in the BM [6]–[8]. Humans with mutations in *DAG1* have cognitive impairment and mild myopathy [9],[10]. Alpha-Dystroglycan is heavily glycosylated and these post-translational modifications are critical for binding of alpha-Dystroglycan to BM ligands and BM deposition [6],[11]. Mutations in six genes required for normal glycosylation of alpha-Dystroglycan have been identified thus far, and the resultant diseases are commonly referred to as the dystroglycanopathies (reviewed in [12]). These diseases show a wide clinical spectrum with only modest correlation between genotype and clinical phenotype [12],[13]. Screening patient populations suggests that there are as yet unidentified genes involved in dystroglycanopathies [14]. There are zebrafish orthologues of all six dystroglycanopathy genes [15],[16]. Morpholinos (MOs) against two of these genes have been characterized, and both lead to muscle degeneration (Fukutin related protein Q0PIP5_DANRE, LARGE 2 LARG2_DANRE) [16],[17]. Thus, the zebrafish system may provide a genetically and embryologically accessible model to complement investigations in mammalian model systems.

Both the DGC and integrin alpha7beta1 heterodimers contribute to force production in mouse muscle, but only the DGC is required to maintain the attachment between the muscle cell membrane (sarcolemma) and the surrounding BM during lengthening contractions [18]. Integrin alpha7 (E7FGC7_DANRE), in contrast, functions primarily at MTJs [19],[20]. Despite their differing roles in sarcolemma-BM attachment, these two receptor complexes can partially compensate for one another [21]–[23], and mutations in components of both receptor complexes greatly exacerbate the extent of muscle atrophy in mice [24],[25]. These data suggest that dynamic cell-ECM signaling mediates adaptive responses when normal muscle architecture is perturbed. Thus, one therapeutic approach is to enhance the intrinsic compensatory relationships between cell adhesion proteins and their ECM microenvironment by potentiating the adhesion of the alternate complex to the BM.

Vertebrate muscle is derived from somites, segmentally reiterated structures delineated by the formation of somite boundaries. As development proceeds, a subset of somitic cells generate muscle, and the somite boundary gives rise to the myotendinous junction (MTJ), which is the major site of force transmission from muscle to the skeletal system. In zebrafish, elongation of fast-twitch muscle fibers and their subsequent attachment to the MTJ correlates with an increase in laminin polymerization at the MTJ [26]. We have identified a novel cell adhesion pathway required for laminin polymerization at the MTJ in vivo. We found that Nicotinamide riboside kinase 2b (Nrk2b Q7ZUR6_DANRE)-mediated NAD+ synthesis potentiates laminin polymerization and subcellular localization of paxillin (Q6R3L1_DANRE), an integrin-associated adaptor protein [27]. Yeast and human Nrks function in an alternative salvage pathway that generates Nicotinamide Adenine Dinucleotide (NAD+) [28],[29]. Exogenous NAD+ rescues MTJ morphogenesis in Nrk2b-deficient zebrafish embryos, indicating that zebrafish Nrk2b also functions to generate NAD+. Our previous results showing a requirement for Nrk2b-generated NAD+ in initial BM morphogenesis lead us to hypothesize that activation of the Nrk2b pathway, either through chemical (NAD+ supplementation) or gene therapy (paxillin overexpression) approaches, would be sufficient to activate the intrinsic compensation between cell-ECM adhesion proteins and result in a novel method of BM augmentation. Herein we will investigate whether the Nrk2b pathway can be exploited to augment laminin polymerization and thus improve muscle tissue structure and function in dystrophic embryos.

Zebrafish deficient for Dag1 display progressive muscle atrophy [30]. Here, we show that muscle atrophy is preceded by degeneration of the MTJ BM. As we previously showed that Nrk2b-mediated NAD+ synthesis is necessary for MTJ BM organization during development; we hypothesized that exogenous NAD+ would improve MTJ BM organization in *dag1* morphants. Indeed, supplementation of *dag1* morphants with NAD+ or a vitamin precursor of NAD+ significantly improved muscle structure. Muscle function was also improved: NAD+- or Emergen-C-supplemented *dag1* morphants swam faster after a touch stimulus. Nrk2 interacts with the cytoplasmic tail of integrin beta1 in the integrin alpha7beta1 complex [31]. Surprisingly, exogenous NAD+ reduced muscle degeneration in *integrin alpha7* (*itga7*) morphants, indicating that Itga7 is not the only integrin in the Nrk2b pathway. We find that the mechanism of action of zebrafish Nrk2b involves a different integrin receptor for laminin, integrin alpha6 (Itga6 A8WHQ8_DANRE). The intracellular cell-ECM adhesion protein paxillin is downstream of Nrk2b-mediated NAD+ synthesis during MTJ development [27]. Paxillin overexpression was sufficient to improve laminin organization and significantly reduce muscle defects in *dag1* morphants. Taken together, these results suggest manipulation of NAD+ precursors/biosynthetic enzymes and paxillin as new potential therapeutic approaches for treatment of not only muscle diseases but also diseases/syndromes that affect laminin integrity.

## Results

### NAD+ Supplementation Reduces Muscle Degeneration in *dystroglycan* (*dag1*) Morphants

**Figure 1:**
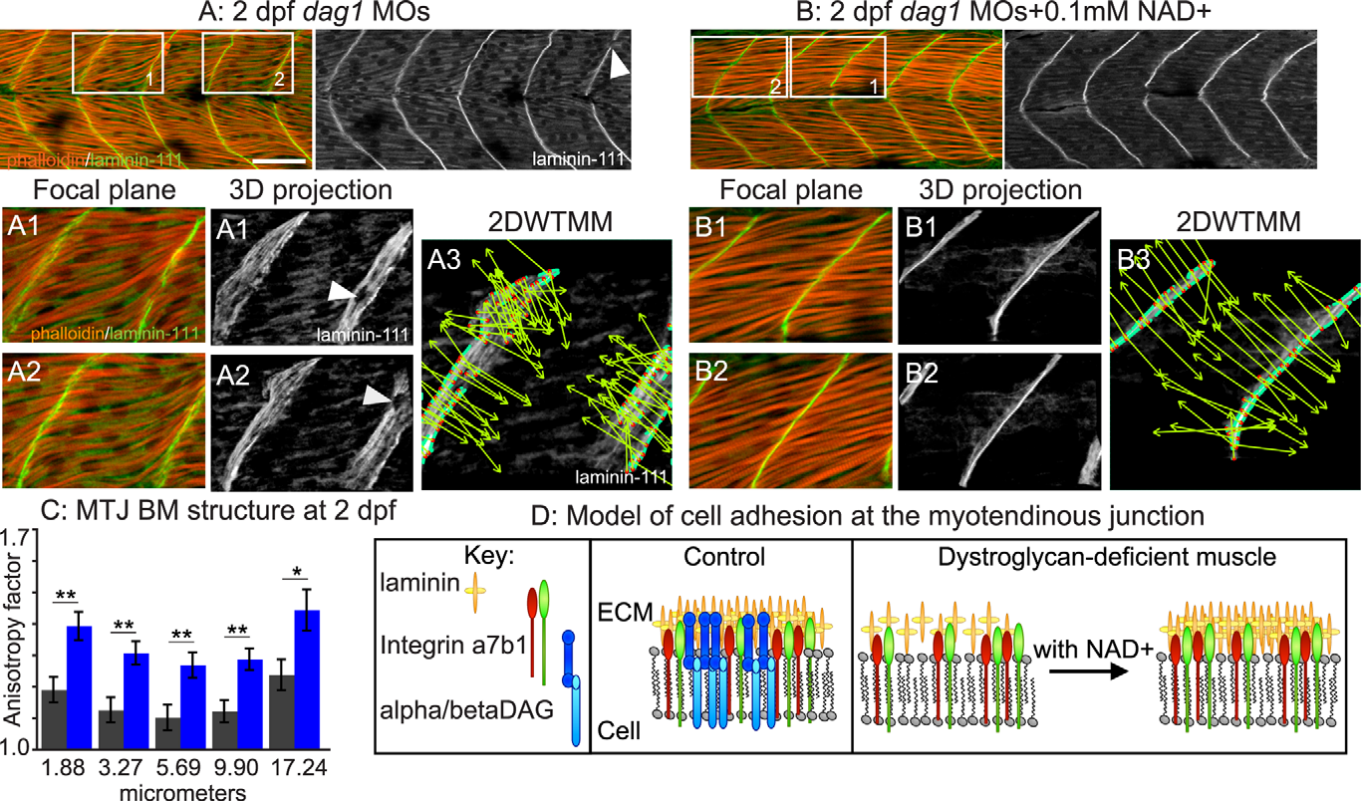
Exogenous NAD+ improves the structure of muscle in *dag1* morphants. (A–B) Anterior left, dorsal top, side-mounted 2 dpf embryos stained for actin (phalloidin, red) and laminin-111 (green or white). Qualitatively, laminin-111 antibody staining appears to be within myotomes and less well aligned at the MTJ BM in *dag1* morphants (A) compared to *dag1* morphants treated with 0.1 mM NAD+ (B). White boxes in (A) and (B) correspond to numbered panels below. White arrowheads indicate holes in the MTJ BM. (A3, B3) 2DWTMM analysis of laminin-111 stained *dag1* morphants (A3) and NAD+-supplemented *dag1* morphants (B3). Maxima nodes are in red, maxima chains are in blue, and vectors pointing in the direction of the maximum intensity gradient are in green. Parallel vectors reflect greater organization. (C) Quantification of the anisotropy factor. The anisotropy factor is the sum of the vector angles. A greater anisotropy factor denotes more organization. NAD+ treatment of *dag1* morphants (blue bars) significantly increases organization of laminin-111 compared to *dag1* morphants (gray bars) over multiple size scales; *p<0.05, **p<0.01. (D) Model of the MTJ. Transmembrane receptors, integrins, and the DGC bind extracellular laminin. In *dag1* morphants, laminin is less organized at the MTJ BM. Exogenous NAD+ improves laminin organization in the MTJ BM in Dag1-deficient zebrafish. Scale bar is 50 micrometers.

Dystroglycan null mice die around the time of implantation [32]. In contrast, zebrafish *dag1* is maternally expressed, Dag1-deficient zebrafish gastrulate, and initial muscle fiber development is normal [30]. Staining for laminin-111 in the ECM microenvironment of Dag1-deficient zebrafish muscle showed that initial BM development is also normal at 1 day post-fertilization (dpf) [27]. At this point in time, laminin-111 is thought to be the major laminin heterotrimer present [33]. Given the critical role of the ECM microenvironment in maintaining fiber integrity, we asked whether the MTJ BM degenerates over time. Although laminin concentrated at the MTJ BM in 2 dpf *dag1* morphants, staining intensity was variable (white arrowhead, Figure 1A). 3-D reconstruction of laminin-111 highlighted inconsistencies in the MTJ BM in *dag1* morphants (Figure 1A1–A2; white arrowheads show holes in the MTJ BM). Because we have shown that Nrk2b-mediated NAD+ synthesis is *necessary* for normal organization of the MTJ BM at 1 dpf, we asked whether exogenous NAD+ would be *sufficient* to improve MTJ BM structure at 2 dpf in *dag1* morphants. Qualitatively, MTJ BM structure appeared to be better aligned in the medial-lateral dimension in *dag1* morphants supplemented with NAD+ compared to untreated *dag1* morphants (Figure 1B, laminin-111 panels). To quantify these observations, we used a mathematical formalism, the 2DWTMM (2-dimensional wavelet transform modulus maxima) [27],[34]–[36], to interrogate MTJ BM structure. The MTJ BM was significantly more organized in NAD+-supplemented *dag1* morphants compared to untreated morphants. This increased organization is visually represented by the parallel alignment of vectors oriented in the direction of the maximal intensity gradient (Figure 1A3, B3). The anisotropy factor (a readout of organization) is derived from the sum of the vectors and is significantly higher in NAD+-supplemented *dag1* morphants (Figure 1C, **p<0.01, *p<0.05). Therefore, NAD+ supplementation increases organization of the BM in *dag1* morphants.

**Figure 2:**
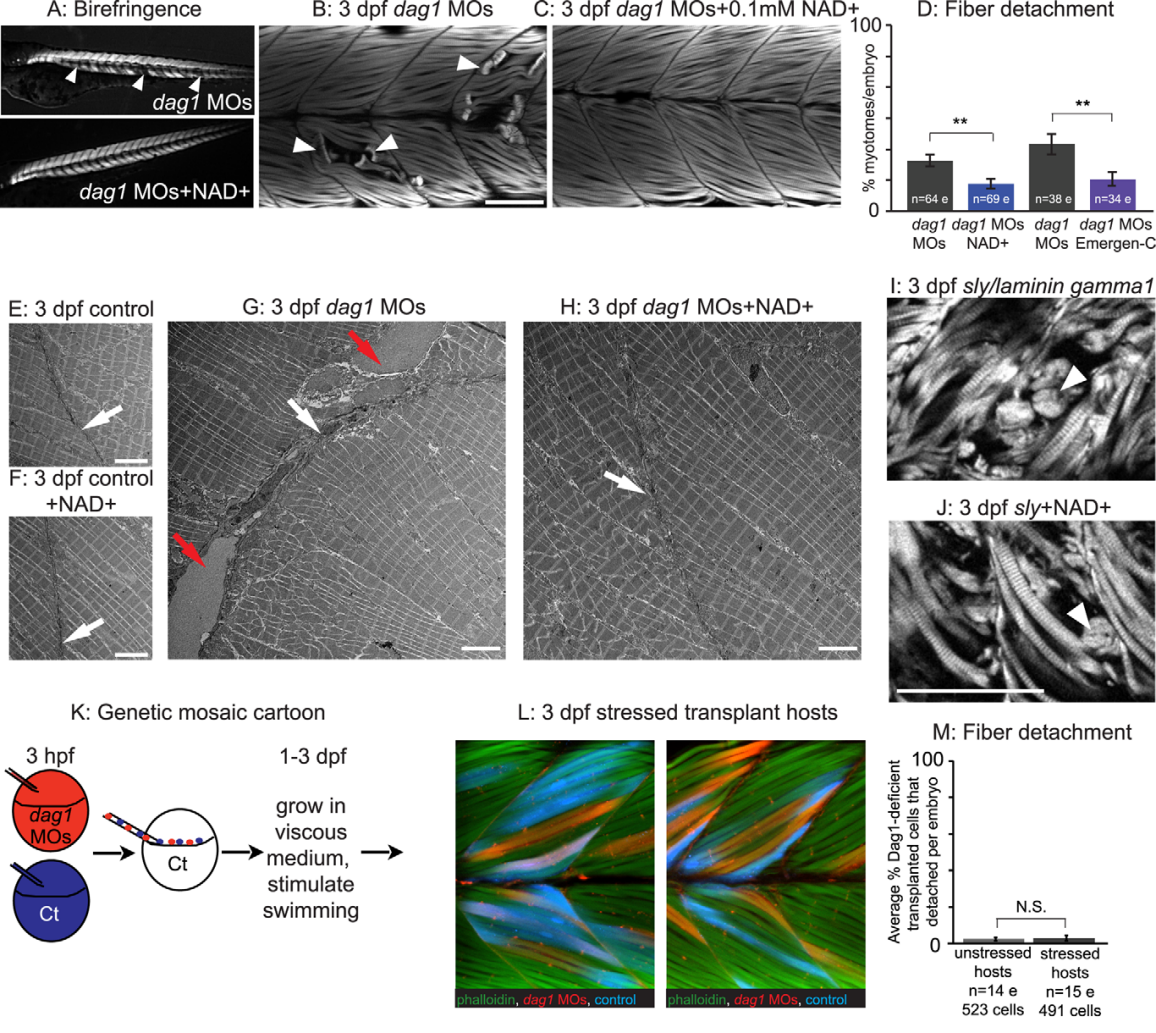
An organized ECM microenvironment rescues fiber resiliency in Dag1-deficient cells. (A) Anterior left, dorsal top, side-mounted, 3 dpf embryos. Polarized light microscopy shows loss of birefringence in *dag1* morphant myotomes (white arrowheads). Birefringence is rescued in NAD+-supplemented *dag1* morphants. (B, C, I, J, L) Anterior left, dorsal top, side-mounted, 3 dpf embryos stained with phalloidin (white or green). Fiber detachment is readily observed in *dag1* morphants (B, white arrowheads), whereas *dag1* morphants supplemented with NAD+ display less fiber detachment (C). (D) Compared to *dag1* morphants (gray bars), NAD+ supplementation (blue bar) and vitamin supplementation with Emergen-C (purple bar) significantly reduce fiber detachment; **p<0.01. (E–H) Transmission electron micrographs showing normal BMs (white arrows) and disrupted BMs (red arrows). (I) The dystrophic phenotype of 3 dpf *sly/laminin gamma1* mutant zebrafish. White arrowhead points to detached fibers. (J) NAD+ does not rescue the dystrophic phenotype in *sly* mutants, suggesting that NAD+-mediated amelioration of dystrophy requires laminin. (K) Genetic mosaic cartoon depicting transplantation of fluorescent dextran-labeled *dag1* morphant (red) and control (blue) cells into unlabeled, control hosts. Some embryos were stressed (frequently stimulated to swim in a viscous medium), and all hosts were reared to 3 dpf. (L) Transplanted control cells (blue) and *dag1* morphant cells (red) remain attached to MTJs, even when hosts are stressed. This suggests that a normal host ECM microenvironment is sufficient for resiliency of *dag1* morphant cells and supports that NAD+ functions via augmentation of the ECM microenvironment. (M) The vast majority of Dag1-deficient cells remain attached in unstressed (513/523 Dag1-deficient cells were attached) and stressed hosts (479/491 Dag1-deficient cells were attached), N.S., not significant. Scale bars are 50 micrometers in (B) and (J) and 5 micrometers in (E–H).

It is not known if detachment of muscle fibers from the MTJ BM contributes to human muscular dystrophies because biopsies are excised from the musculature to avoid injury to the tendon, but MRI studies do suggest that muscle damage is more severe closer to the MTJ [37],[38]. In dystrophic zebrafish, muscle fibers detach from the MTJ prior to apoptosis in *laminin alpha2* [39], *laminin beta2* [4], and *dag1* mutants [17], implicating failure of muscle fiber-MTJ adhesion as the primary etiology in these models of muscular dystrophy. We hypothesized that the increased organization of the MTJ BM in NAD+-supplemented Dag1-deficient zebrafish would reduce the frequency of muscle fiber detachment. The ordered array of myofibers in wild-type skeletal muscle results in birefringence of polarized light [40]. Muscle degeneration in *dag1* morphants results in gaps in birefringence (Figure 2A, white arrowheads). Birefringence was improved in NAD+-supplemented *dag1* morphants (Figure 2A). We next used phalloidin staining to visualize detached muscle fibers and quantitatively assess muscle degeneration. The percent of muscle segments per embryo with detached muscle fibers was significantly reduced in *dag1* morphants supplemented with NAD+ compared to untreated *dag1* morphants (Figure 2B–D; white arrowheads indicate detached fibers; **p<0.01). These data show that NAD+ is sufficient to improve birefringence and reduce detachment of muscle fibers.

NAD+ is synthesized via multiple different pathways. Nrk2 is part of an alternative salvage pathway that mediates NAD+ biosynthesis [28],[29]. In addition, Tryptophan and vitamin B3 (niacin, niacinamide, nicotinamide, or nicotinic acid) are NAD+ precursors. We next asked whether vitamin supplementation would increase NAD+ levels in vivo. We choose Emergen-C packets for vitamin B3 supplementation because they (1) are soluble in Embryo Rearing Medium (ERM) and do not disrupt development (unpublished data) and (2) contain 5 mg niacin. As shown in Figure 2D, addition of Emergen-C significantly reduced muscle degeneration in *dag1* morphants compared to nonsupplemented morphants. This result implicates the complex of B vitamins in Emergen-C as potent precursors of NAD+ in zebrafish and in promoting muscle health.

In addition to using the 2DWTMM, polarized light, and phalloidin staining assays to visualize and quantify changes in muscle tissue structure, we also utilized transmission electron microscopy. Electron microscopy showed that NAD+ supplementation did not affect muscle structure in control embryos (Figure 2E–F) but improved structure in *dag1* morphants. Electron microscopy revealed deficiencies in the MTJ of *dag1* morphant muscle. Abnormal gaps in the MTJ BM were observed (Figure 2G, red arrows). In NAD+-supplemented *dag1* morphants, BM structure was improved (Figure 2H, white arrow).

### A Normal MTJ BM Microenvironment Is Sufficient to Rescue the Structure of Dystrophic Muscle

NAD+ supplementation significantly increases MTJ BM organization *prior* to the onset of dystrophy. This result suggests that the mechanism of action of NAD+ could be through BM augmentation. If NAD+ supplementation reduces the incidence of dystrophy via laminin augmentation, NAD+ would not protect against muscle degeneration in *laminin* mutant zebrafish. We asked if laminin is required for NAD+-mediated amelioration of dystrophy by utilizing *laminin gamma1* mutant zebrafish, which display muscle degeneration starting at 3 dpf (Figure 2I, laminin gamma1 UniProtKB accession number Q1LVF0 LAMC1_DANRE). *laminin gamma1* mutants supplemented with exogenous NAD+ showed no improvement in the incidence of dystrophy compared to untreated mutants (Figure 2I–J). This result shows that laminin is required for NAD+-mediated reduction of fiber detachment and supports our hypothesis that the protective effects of NAD+ occur through BM augmentation. This result also aligns with our previous finding that NAD+ reduces MTJ failure in 2 dpf *nrk2b* morphants but not *laminin beta1* morphants [27]. Together, these results strongly suggest that the protective effects of NAD+ supplementation on MTJ failure and fiber detachment result from augmentation of laminin in the BM.

We used genetic mosaic analysis to further test our hypothesis. If the DGC is required cell autonomously for maintenance of muscle fiber adhesion, we would predict that *dag1* morphant cells in control hosts would degenerate as observed in *dag1* morphants. If, however, a normal BM provides a supportive environment for muscle fibers, we would predict that *dag1* morphant cells would be less likely to degenerate in control embryos. To discriminate between these two outcomes, we transplanted fluorescent dextran-labeled *dag1* morphant cells into control embryo hosts at the blastula stage, grew the embryos until 3 dpf, and asked whether *dag1* morphant cells were viable (Figure 2K). The vast majority of *dag1* morphant cells were viable (Figure 2L). Only 10 out of 523 transplanted *dag1* morphant cells (1.9%) detached from the MTJ BM. The incidence of fiber detachment per embryo in transplant hosts was significantly less than in *dag1* morphant embryos (*p*<0.001). In addition, *dag1* morphant cells fused with fluorescent dextran-labeled control cells (Figure 2L, fusion indicated by pink cells). A more rigorous test of our hypothesis would be to ask whether Dag1-deficient cells are more likely to detach in control hosts when muscle tissue is stressed. We repeatedly stimulated transplant host embryos to swim through a viscous medium as described previously [39]. Of the 491 Dag1-deficient cells analyzed in 15 stressed host embryos, only 12 cells (2.4%) detached from the MTJ. Although a higher percentage of morphant cells detached in stressed hosts versus nonstressed hosts, the incidence of dystrophy per embryo did not significantly differ depending on stress (Figure 2M, *p* = 0.81) and was still significantly different than in *dag1* morphants (*p*<0.001). These data clearly indicate that local integrity of the ECM microenvironment is sufficient for muscle cell adhesion and that Dag1 is not required cell autonomously for maintenance of this adhesion.

### Itga7 Is Required for Nrk2b-NAD+-Laminin-Mediated Reduction of Dystrophy in *dag1* Morphants

**Figure 3:**
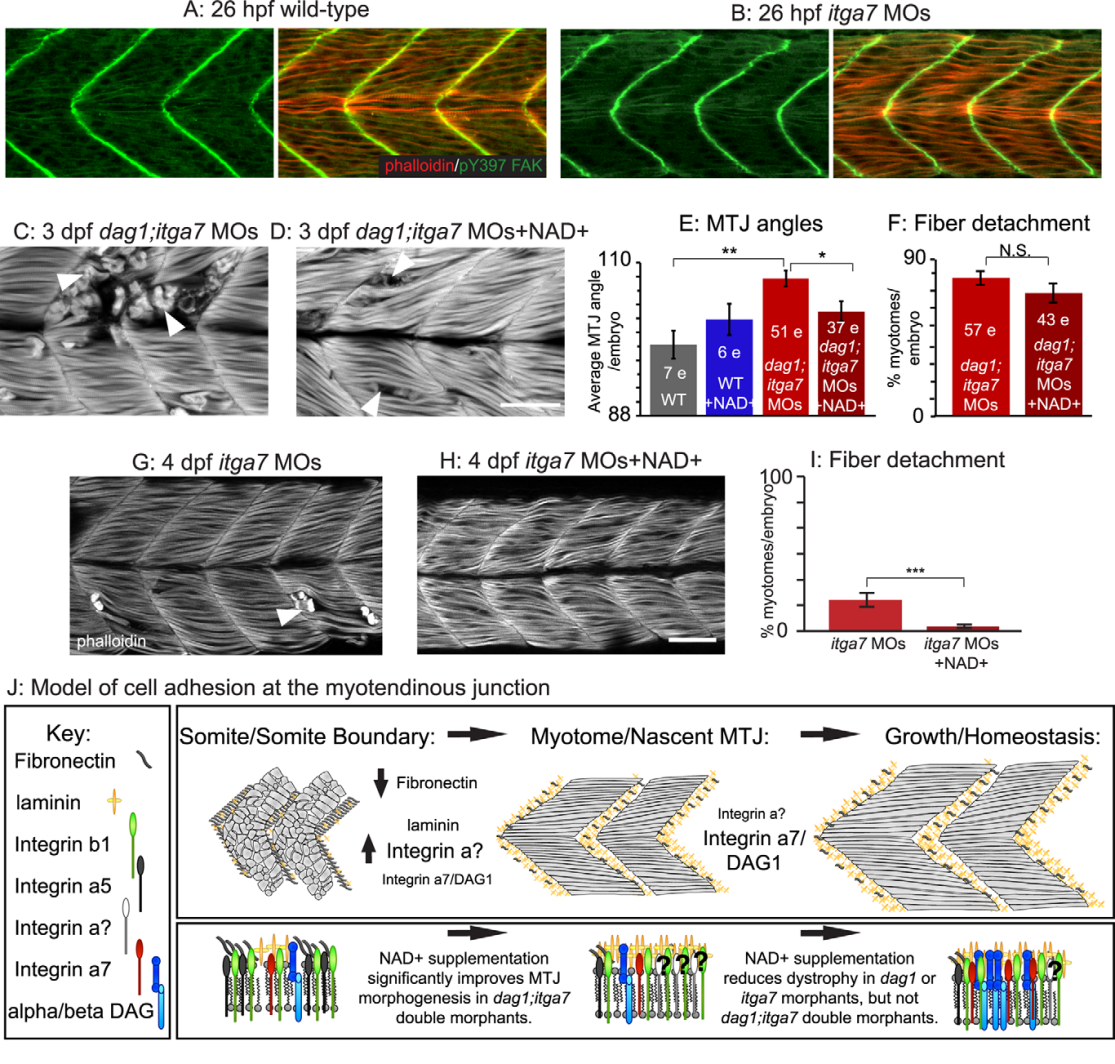
Itga7 is required for NAD+-mediated reduction of fiber degeneration in *dag1* morphants. (A–D, G–H) Anterior left, dorsal top, side-mounted embryos stained with phalloidin (red or white) or pY397 FAK (green). (A–B) Muscle morphogenesis proceeds normally in *itga7* morphants. Phosphorylated FAK (green) outlines fibers and concentrates at the MTJ, and actin distribution in slow- and fast-twitch muscle fibers (red) appears normal in 26 hpf *itga7* morphants compared to wild-types. (C) 3 dpf *dag1;itga7* double morphant. (D) 3 dpf NAD+-supplemented *dag1;itga7* double morphant. MTJ morphogenesis is disrupted in *dag1;itga7* double morphants as displayed by wider MTJ angles (C). MTJ morphogenetic defects were rescued by NAD+ in *dag1;itga7* double morphants (D), suggesting that another laminin receptor is sufficient for NAD+-mediated MTJ improvements. (E) Quantification of MTJ angles shows that *dag1;itga7* double morphants have significantly wider MTJ angles than wild-types, and NAD+ significantly reduces this defect; **p<0.01, *p<0.05. (F) Quantification of incidence of dystrophy per embryo shows no significant difference in *dag1;itga7* double morphants upon addition of exogenous NAD+, suggesting that Itga7 is required for NAD+-mediated reduction of dystrophy in *dag1* morphants; N.S., not significant. However, fewer fibers appeared to detach in *dag1;itga7* double morphants supplemented with NAD+ (D), again suggesting the involvement of another receptor for laminin in NAD+ action. (G–H) Mild fiber detachment is readily observed in 4 dpf *itga7* morphants (G) and reduced in NAD+-treated *itga7* morphants (H). (I) NAD+ treatment significantly decreases fiber degeneration in *itga7* morphants; ***p<0.001. (J) Model of cell adhesion at the MTJ. The transition from a somite boundary to a MTJ involves the downregulation of Fibronectin and the upregulation of laminin and laminin receptors. Our results suggest that laminin receptors, Itga7 and Dag1, play a role in this transition, but the primary receptor involved is an unknown integrin. In maintenance of fiber adhesion at the MTJ, our data show that either Itga7 or Dag1 is required, but also suggest the involvement of an additional laminin receptor. Scale bar is 50 micrometers.

The two “canonical” laminin receptors in muscle tissue are the DGC and integrin alpha7beta1 receptor complexes. Itga7 is disrupted in CMD with integrin deficiency [41]. As the DGC is not required for Nrk2b-NAD+-laminin-mediated reduction of dystrophy (Figure 2), we asked if Itga7 is required for the amelioration of dystrophy in *dag1* morphants by exogenous NAD+. A zebrafish model of CMD with integrin deficiency has been generated [19],[42]. Muscle degeneration in *itga7* morphants is apparent at approximately 4.5 dpf. One critical axis to consider when evaluating muscle homeostasis is whether initial muscle morphogenesis is disrupted. It is not known if initial muscle development proceeds normally in *itga7* morphants. We analyzed muscle morphogenesis at 26 hpf and found that both MTJ and muscle fiber development appeared normal (Figure 3A–B). We next generated *dag1;itga7* double morphant embryos by co-injecting half the functional doses of *dag1* and *itga7* MOs. Surprisingly, given that early MTJ morphogenesis appears grossly unaffected in single morphants, MTJ morphogenesis was disrupted in double morphants. MTJ angles were abnormally wide (Figure 3C,E), similar to *laminin beta1* and *gamma1* mutant embryos [43]. This disruption in MTJ development is dramatically greater than would be predicted based upon the individual phenotypes and suggests Dag1- and Itga7-mediated adhesion to laminin is synergistically required for MTJ morphogenesis. This was surprising given that MTJ morphogenesis appears normal in single morphants. We next asked whether exogenous NAD+ would rescue initial MTJ morphogenesis in *dag1;itga7* morphants (Figure 3D). We found that MTJ angles in NAD+-supplemented *dag1;itga7* morphants were significantly different from untreated double morphants (Figure 3E, 103.03±1.41 degrees versus 107.74±1.16 degrees, respectively; **p<0.01, *p<0.05). Thus, NAD+ supplementation is sufficient to rescue early morphogenetic events that require adhesion to laminin in a Dag1- and Itga7-independent manner.

The above data indicate that Itga7 and Dag1 are synergistic with regards to initial muscle development, but NAD+ supplementation is sufficient to improve MTJ development in *itga7;dag1* double morphant embryos. We next asked whether Itga7 is required for NAD+-mediated reduction of dystrophy in *dag1* morphants. We found that NAD+ supplementation was not sufficient to significantly reduce the percent of muscle segments with fiber detachment in double morphants (Figure 3F, *p* = 0.2). However, qualitatively, fewer fibers per myotome were detached (compare Figure 3C to 3D; we were unable to reliably count detached fibers in myotomes with severe degeneration and thus could not quantitate this observation). Taken together, these data indicate that Itga7 is required for NAD+-mediated reduction of dystrophy in *dag1* morphants and that NAD+ supplementation is not sufficient to reduce the frequency of myotomes with muscle degeneration when both canonical laminin receptors (Dag1, Itga7) are compromised.

The above data clearly show that Itga7 plays a role in NAD+-mediated reduction of dystrophy in *dag1* morphants. Interestingly, two results suggest that an additional laminin receptor participates in muscle development and homeostasis: (1) NAD+ supplementation rescues early MTJ morphogenesis in *dag1*;*itga7* double morphants, and (2) overall muscle structure is qualitatively improved with NAD+ supplementation in *dag1;itga7* double morphants. A key experiment to clarify whether an additional laminin receptor also participates in the Nrk2b-NAD+-laminin pathway is to ask whether NAD+ supplementation is sufficient to reduce dystrophy in *itga7* morphants. NAD+ supplementation significantly reduced the incidence of dystrophy in *itga7* morphants (Figure 3G–I; ***p<0.001). This result indicates that there is an additional, “non-canonical” laminin receptor that mediates laminin signaling downstream of NAD+ during musculoskeletal homeostasis.

### The Nrk2b-NAD+-Laminin Pathway Involves the Itga6 Receptor

**Figure 4:**
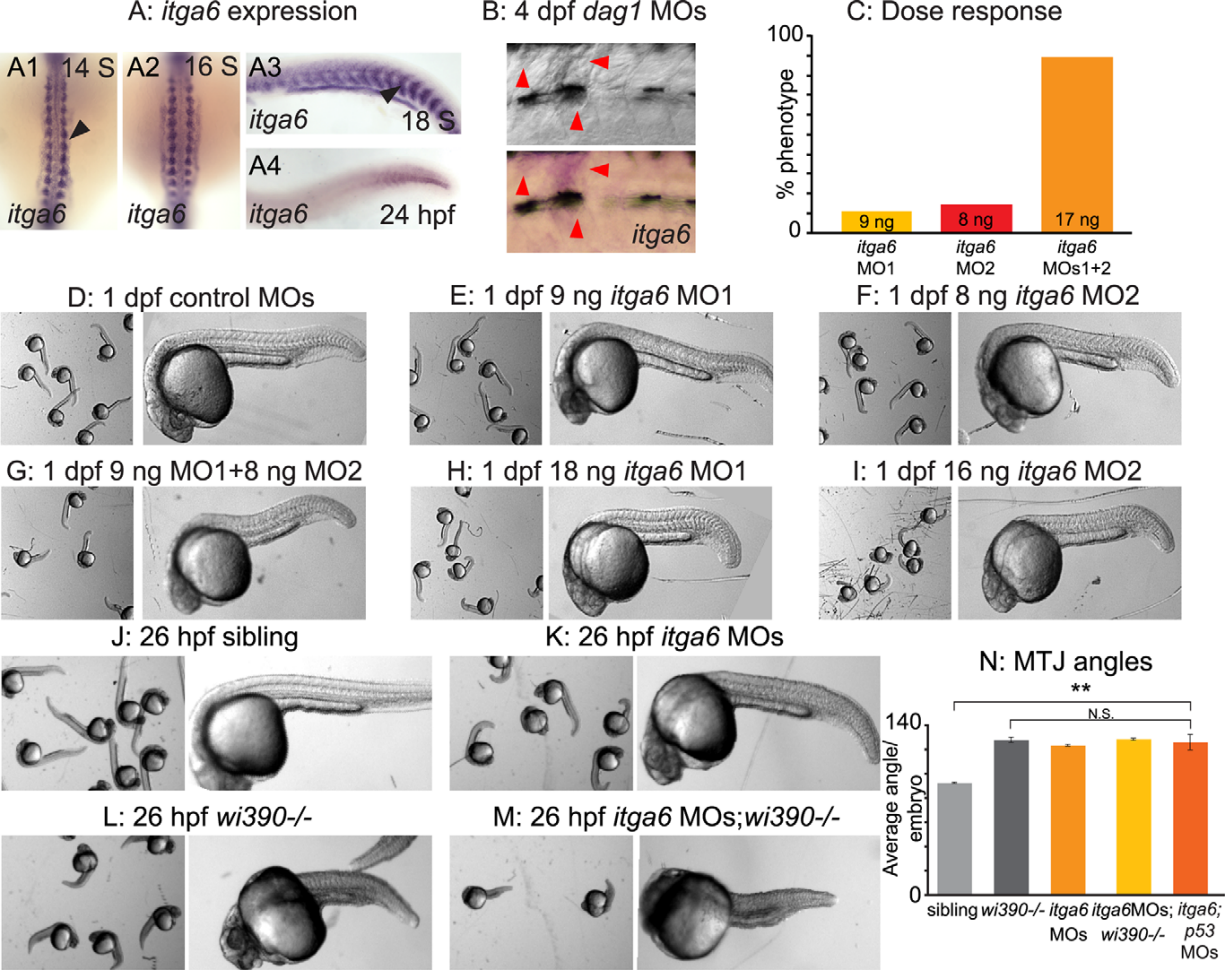
*itga6* is upregulated in regenerating muscle and characterization of *itga6* MOs. (A) In situ hybridizations showing *itga6* expression (purple). (A1–2) Dorsal view, anterior top. (A3–4, B) Side view, anterior left, dorsal top. (A1–4) Black arrowheads denote somitic expression. *itga6* expression is high during early muscle development, then decreases. (B) *itga6* is re-expressed in regenerating muscle. *itga6*, not normally expressed in muscle at 4 dpf, is observed in dystrophic lesions of 4 dpf *dag1* morphants (red arrowheads). (C) *itga6* MO characterization. Dose response graph. MO1 and MO2 generate the same phenotype and synergize when co-injected. (D–M) Brightfield images, side view, anterior left, dorsal top, 1 dpf embryos. (D–I) Phenotypic analysis of *itga6* MOs 1 and 2. Embryos injected with low doses of MO1 (E) or MO2 (F) are morphologically similar to controls (D). Combining the two lower doses of MOs 1 and 2 results in a truncated body axis with myotomes that are narrower in the anterior-posterior dimension (G). The identical phenotype is obtained when higher doses of either MO1 (H) or MO2 (I) are injected. (J–M) Pseudo-genetic epistasis analysis. (J) Siblings, (K) *itga6* morphants, (L) *wi390*−/−/*laminin gamma1* mutants, and (M) *itga6* MOs;*wi390*−/−. Note that injection of *itga6* MOs into *laminin* mutants does not change their phenotype, suggesting Itga6 functions in laminin signaling and adhesion. (N) Average MTJ angles of 1 dpf embryos. MTJ angles in morphants, mutants, and morphant/mutants do not significantly differ from one another and are all significantly wider than in sibling controls; **p<0.01; N.S., not significant.

The above data indicate that the primary beneficial effect of NAD+ on muscle architecture is increased organization of laminin in the BM. However, neither of the two laminin receptors known to be required for muscle structure (Dag1, Itga7) are required individually for Nrk2b-NAD+-laminin mediated reduction of dystrophy (Figures 2 and 3). In order to identify mechanisms downstream of laminin, we focused on identifying the relevant laminin receptor. The laminin-binding family of integrin alpha chains that partner with integrin beta1 includes alpha3, alpha6, and alpha7 [44]. Only two of these chains, *itga6* and *itga7*, are expressed during zebrafish muscle development ([42], Thisse B. and Thisse C., http://zfin.org; Figure 4A). Expression of *itga6* is high during initial muscle development and then declines. This correlates with the low-level expression of *itga6* in human muscle [45].

Interestingly, *itga6* has recently been implicated in muscle development and regeneration in multiple mammalian species [46]–[49]. We asked whether *itga6* expression is upregulated in *dag1* morphant zebrafish in dystrophic lesions where regeneration is occurring. As in other vertebrates, *itga6* expression was low in zebrafish muscle but clearly visible in dystrophic lesions (Figure 4B, red arrowheads). Given these recent results, combined with our data showing upregulation of *itga6* in *dag1* morphants, we hypothesized that Itga6 is involved in the Nrk2b-NAD+-laminin pathway. We tested this hypothesis by using morpholino (MO)-mediated knockdown. We first characterized MOs against *itga6*. Injection of varying amounts/combinations of two different, nonoverlapping, translation-blocking MOs showed that both MOs generated the same phenotype and acted synergistically (Figure 4C–I). The *itga6* morphant phenotype was nearly identical to that of *laminin beta1* and *gamma1* mutants and *nrk2b* morphants [27],[36]. This striking phenotypic similarity suggested that *itga6* may function in the Nrk2b-NAD+-laminin pathway. To confirm that Itga6 acts in laminin signaling/adhesion, we conducted pseudo-genetic epistasis analysis. The transcripts for *laminin beta1* and *gamma1* are maternally expressed. Thus, injection of *laminin beta1/gamma1* MOs into *laminin beta1/gamma1* mutants slightly worsens the phenotype [50]. Injection of *itga6* MOs into *laminin gamma1* mutants also slightly worsened the phenotype (Figure 4J–M), but average MTJ angles were not significantly different in *itga6;laminin gamma1* morphant/mutants compared to *laminin gamma1* mutants (Figure 4N). Together, these data indicate that these MOs are specific and do not cause off-target effects [51]. The above results also suggest that Itga6 does participate in laminin signaling/adhesion.

**Figure 5:**
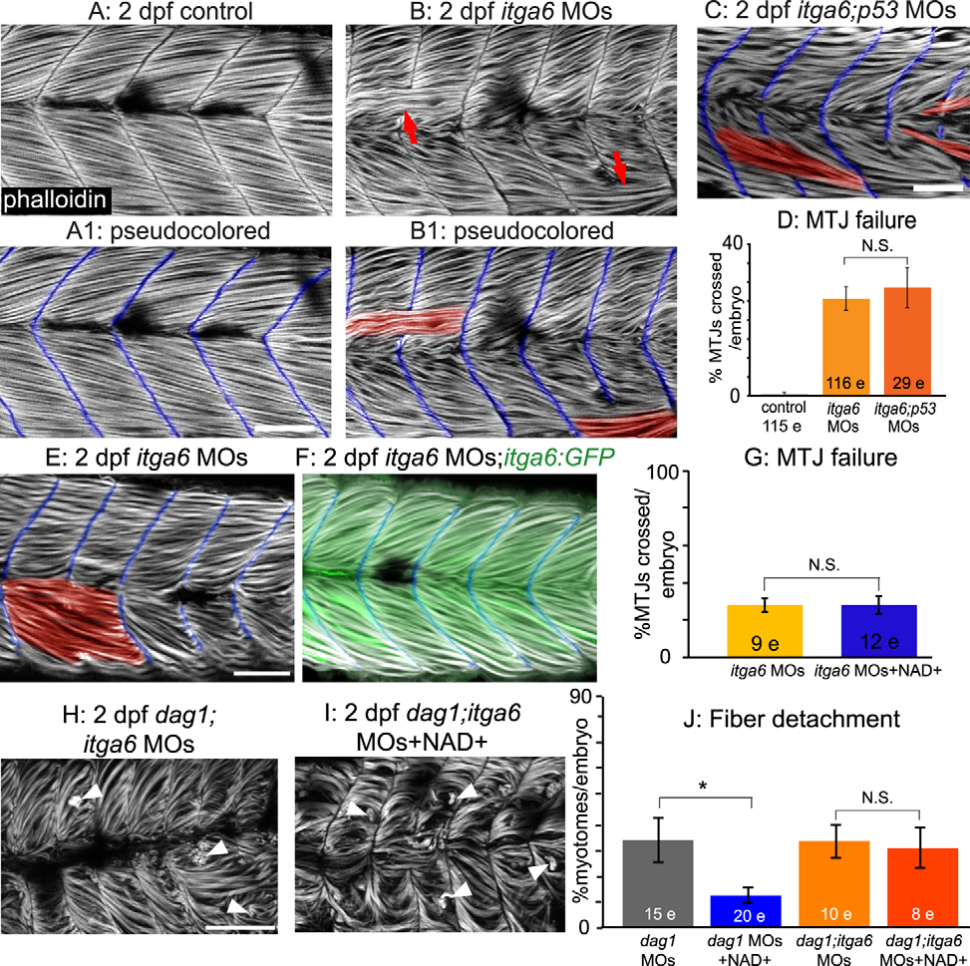
Itga6 functions in the Nrk2b-NAD+-laminin pathway and is required for NAD+-mediated rescue of MTJ morphogenesis and dystrophy. (A–C, E–F, H–I) Anterior left, dorsal top, side-mounted, 2 dpf embryos stained with phalloidin (white) to visualize actin. In pseudocolored panels (A1, B1, C, E, F), MTJ boundaries are blue, and abnormally long muscle fibers are red. (A) MTJs are V-shaped and continuous in control embryos. (B) In *itga6* morphants, MTJs are U-shaped, discontinuous, and crossed by abnormally long muscle fibers (red arrows). (C) Co-injection of *p53* MOs does not rescue MTJ failure in *itga6* morphants. (D) Quantification of MTJ failure at 2 dpf in controls, *itga6* morphants, and *itga6;p53* double morphants. (E–F) Co-injection of *itga6* cDNA that does not contain the MO target sites with *itga6* MOs rescues the *itga6* morphant phenotype. (G) Quantification of MTJ failure shows that NAD+ treatment does not rescue MTJ failure in *itga6* morphants, suggesting that NAD+ requires Itga6 for rescue of MTJ failure. (H) *dag1;itga6* double morphants have U-shaped MTJs and dystrophy (white arrowheads). (I) NAD+ does not reduce MTJ angles (not shown) or dystrophy in *dag1;itga6* double morphants, suggesting that Itga6 is also required for NAD+-mediated rescue of MTJ angles and dystrophy. (J) Quantification of dystrophy shows significant rescue by exogenous NAD+ in *dag1* morphants, but not *dag1;itga6* double morphants; *p<0.05; N.S., not significant. Scale bars are 50 micrometers.

Although somite patterning was relatively normal and somite boundaries formed in these embryos (unpublished data), by 2 dpf approximately 25% of the MTJs had failed (Figure 5B red arrows, 5D). Abnormally long muscle fibers crossed the MTJ at sites of MTJ failure (Figure 5B1, MTJs pseudocolored blue and crossing fibers pseudocolored red). One possible side effect of MOs is that they can activate p53-dependent apoptosis and cause nonspecific cell death [52]. Co-injection with *p53* MO did not alter the phenotype of *itga6* morphants (Figure 5C,D). Co-injection of *itga6* MOs with *itga6* cDNA lacking the MO target sites rescued the morphant phenotype (Figure 5E,F), further suggesting that these *itga6* MOs are specific [51].

NAD+ functions upstream of laminin polymerization during MTJ development [27]; thus, we hypothesized that *itga6* morphants would not be rescued by NAD+ supplementation. Neither exogenous NAD+ nor Emergen-C (unpublished data) were sufficient to rescue MTJ failure at 2 dpf in *itga6* morphants, indicating that Itga6 functions in this pathway downstream of NAD+ (Figure 5G; *p* = 0.99 for NAD+). Although *itga6* morphants were slightly dystrophic (unpublished data), the frequency of dystrophy was too rare to readily ask whether NAD+ supplementation is sufficient to reduce dystrophy in *itga6* morphants. Taken together, these data suggest that Itga6 functions in this pathway downstream of NAD+.

### Itga6 Is Required for NAD+-Mediated Amelioration of Dystrophy in *dag1* Morphants

Given the requirements for Itga6 in muscle development in mouse [46] and our data showing that Itga6 is required in zebrafish muscle development, we asked whether Itga6 is required to mediate reduction of dystrophy in *dag1* morphants. We co-injected *itga6* and *dag1* MOs and assayed the incidence of dystrophy in response to NAD+ supplementation (Figure 5H–I). As with co-injection of *itga7* and *dag1* MOs, initial MTJ morphogenesis was disrupted: myotomes were narrower in the anterior-posterior dimension and wider in the medial-lateral dimension. In contrast to *dag1;itga7* double morphants, NAD+ supplementation was not sufficient to improve MTJ morphogenesis in *dag1;itga6* double morphants (unpublished data; average MTJ angle for *dag1;itga6* morphants, 143.98±3.08 degrees; average MTJ angle for NAD+-treated *dag1;itga6* morphants, 139.50±3.52 degrees, *p* = 0.35). This result indicates that Itga6 is the laminin receptor required for the role of NAD+ in MTJ morphogenesis. NAD+ supplementation also did not ameliorate muscle degeneration in *dag1;itga6* double morphants (Figure 5J, *p* = 0.78). This result indicates that Itga6 is also required for NAD+-mediated amelioration of dystrophy in *dag1* morphants.

### Paxillin Overexpression Is Sufficient to Increase MTJ BM Structure and Decrease Fiber Detachment in *dag1* Morphants

**Figure 6:**
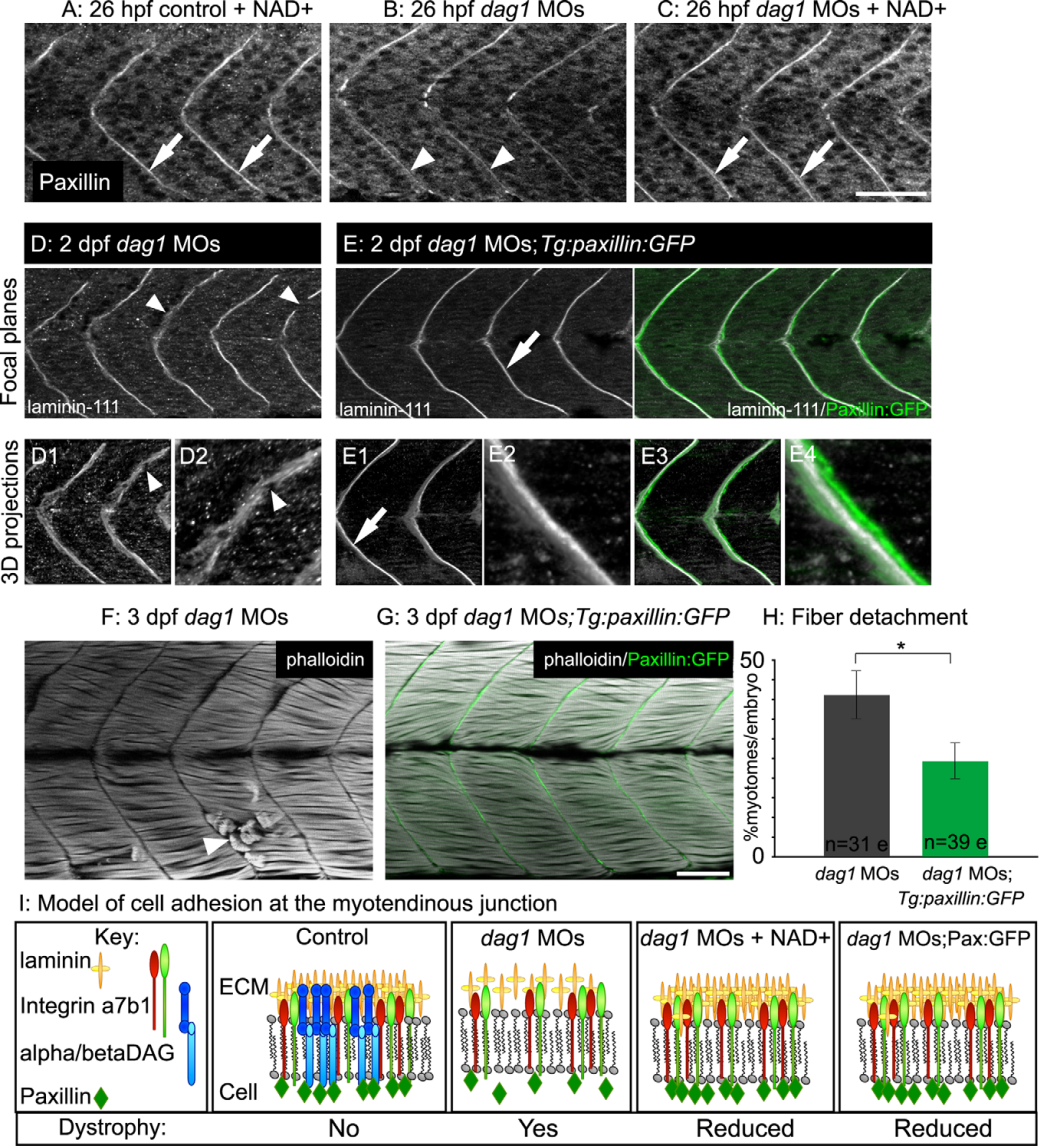
Paxillin overexpression increases laminin organization and ameliorates dystrophy in *dag1* morphants. (A–C) Anterior left, dorsal top, side-mounted, 1 dpf embryos antibody stained for paxillin (white). (A) Paxillin concentrates at the MTJ in both untreated (not shown) and NAD+-treated controls (white arrows). (B) Paxillin is less concentrated at the MTJ in *dag1* morphants (white arrowheads). (C) NAD+ rescues the disrupted concentration of paxillin at the MTJ in *dag1* morphants (white arrows). (D–E) Anterior left, dorsal top, side-mounted, 2 dpf embryos stained with laminin-111 antibody (white). Numbered panels are 3-D reconstructions. (D) *dag1* morphant laminin-111 appears within myotomes, and the MTJ BM is poorly aligned medially laterally and contains holes (white arrowheads). (E) In contrast, paxillin overexpression (green) in *dag1* morphants reduces laminin-111 within myotomes and enhances organization of laminin-111 at the MTJ BM (white arrows). (F–G) Anterior left, dorsal top, side-mounted, 3 dpf embryos stained for actin (phalloidin, white). (F) *dag1* morphant with detached fibers (white arrowhead). (G) Transgenic overexpression of paxillin (green) in *dag1* morphants reduces fiber detachment. (H) Paxillin overexpression significantly reduces the frequency of fiber detachment in *dag1* morphants; *p<0.05. (I) Model of cell adhesion at the MTJ in response to Nrk2b pathway activation via exogenous NAD+ or paxillin overexpression. Scale bars are 50 micrometers.

We previously showed that the integrin-associated adaptor protein paxillin functions downstream of Nrk2b-mediated NAD+ synthesis during MTJ development: paxillin overexpression is sufficient to rescue MTJ morphogenesis in *nrk2b* morphants [27]. Thus, likely through “inside-out” signaling via laminin receptors, paxillin is sufficient to affect the ECM microenvironment. We asked whether overexpression of paxillin would be sufficient to augment MTJ BM organization in dystrophic embryos. Paxillin concentration at the MTJ was slightly disrupted in *dag1* morphants at 26 hpf (Figure 6B). NAD+ supplementation improved paxillin concentration at the MTJ in 26 hpf *dag1* morphants (Figure 6C). Next, we overexpressed paxillin in *dag1* morphants by injecting *dag1* MOs into *Tg:paxillin:GFP* embryos. Paxillin:GFP accounted for approximately 66.05%±8% of total paxillin expressed at 2 dpf in *Tg:paxillin:GFP* embryos (unpublished data). Paxillin overexpression increased organization of laminin-111 at the MTJ in 2 dpf *dag1* morphants (compare Figure 6E to 6D; 2DWTMM analysis not shown; *p<0.05 for wavelets from 1.88 to 4.02 micrometers). Paxillin overexpression also significantly reduced the incidence of fiber detachment in 3 dpf Dag1-deficient embryos (Figure 6F–H; *p<0.05).

**Figure 7:**
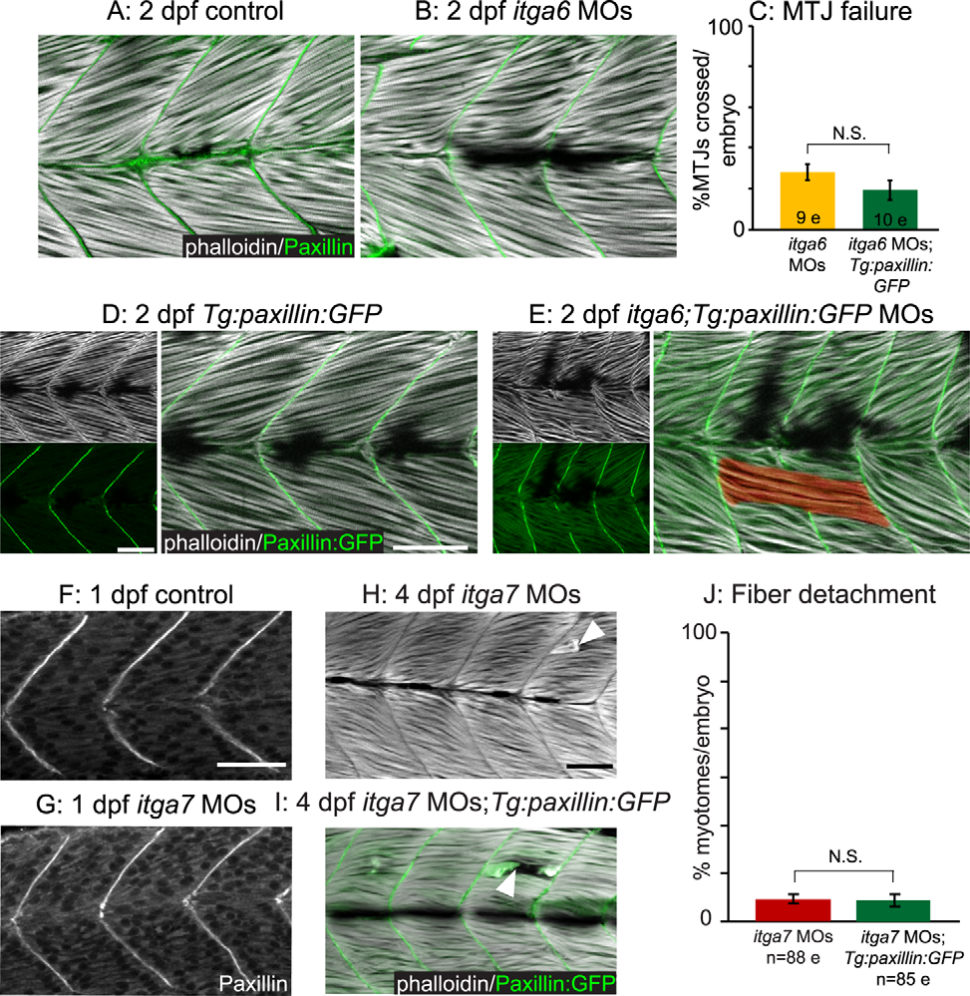
Paxillin action but not subcellular localization requires functional integrin receptors for laminin. (A–B, D–E) Side-mounted, anterior left, dorsal top, 2 dpf embryos stained with phalloidin (white). (A–B) Antibody staining shows that paxillin (green) concentrates at the MTJ in *itga6* morphants (B) as in controls (A). (C) Quantification of MTJ failure shows that paxillin overexpression does not rescue *itga6* morphants. (D–E) Transgenic overexpression of paxillin:GFP (green) does not affect MTJ development in controls (D) and is not sufficient to rescue MTJ failure in *itga6* morphants (E). (F–G) Anterior left, dorsal top, side-mounted, 26 hpf embryos stained for paxillin (white). Paxillin concentrates at the MTJ in *itga7* morphants (G) as in controls (F). (H–I) Side mounted, anterior left, dorsal top, 4 dpf embryos stained with phalloidin (white). Fiber detachment is readily observed in *itga7* morphants (H) and *itga7* morphants transgenically overexpressing paxillin (I, white arrowheads). (J) Paxillin overexpression does not affect fiber detachment frequency in *itga7* morphants. Together, these results suggest that Itga6 and Itga7 are required for paxillin-mediated improvements in muscle tissue. N.S., not significant. Scale bars are 50 micrometers.

As paxillin does not require Dag1 for BM augmentation-mediated reduction of dystrophy (Figure 6), we asked whether integrin receptors are required. We analyzed both the subcellular localization of paxillin upon Itga6 or Itga7 knockdown as well as the ability of transgenic paxillin overexpression to rescue the muscle defects observed in *itga6* or *itga7* morphants. In *itga6* morphants, paxillin concentrated at the MTJ as in control embryos (Figure 7A–B). Paxillin overexpression in *itga6* morphants (Figure 7E) did not reduce the incidence of MTJ failure (Figure 7C; *p* = 0.18). Paxillin also localized to the MTJ in *itga7* morphants (Figure 7G), and paxillin overexpression in *itga7* morphants had no effect on the percent of myotomes per embryo with dystrophic lesions (Figure 7H–J; *p* = 0.9). As paxillin is an integrin adaptor protein, it is perhaps not surprising that paxillin-mediated BM augmentation requires functional integrin receptors for laminin. Our results suggest that Dag1, but not Itga6 or Itga7, is required for localization of paxillin to the MTJ and that the beneficial effects of overexpressing paxillin in Dag1-deficient muscle tissue may result from increasing the amount of MTJ-proximal paxillin involved in inside-out signaling via integrin receptors for laminin.

### NAD+ Supplementation Improves the Mobility of Dystrophic Zebrafish

**Figure 8:**
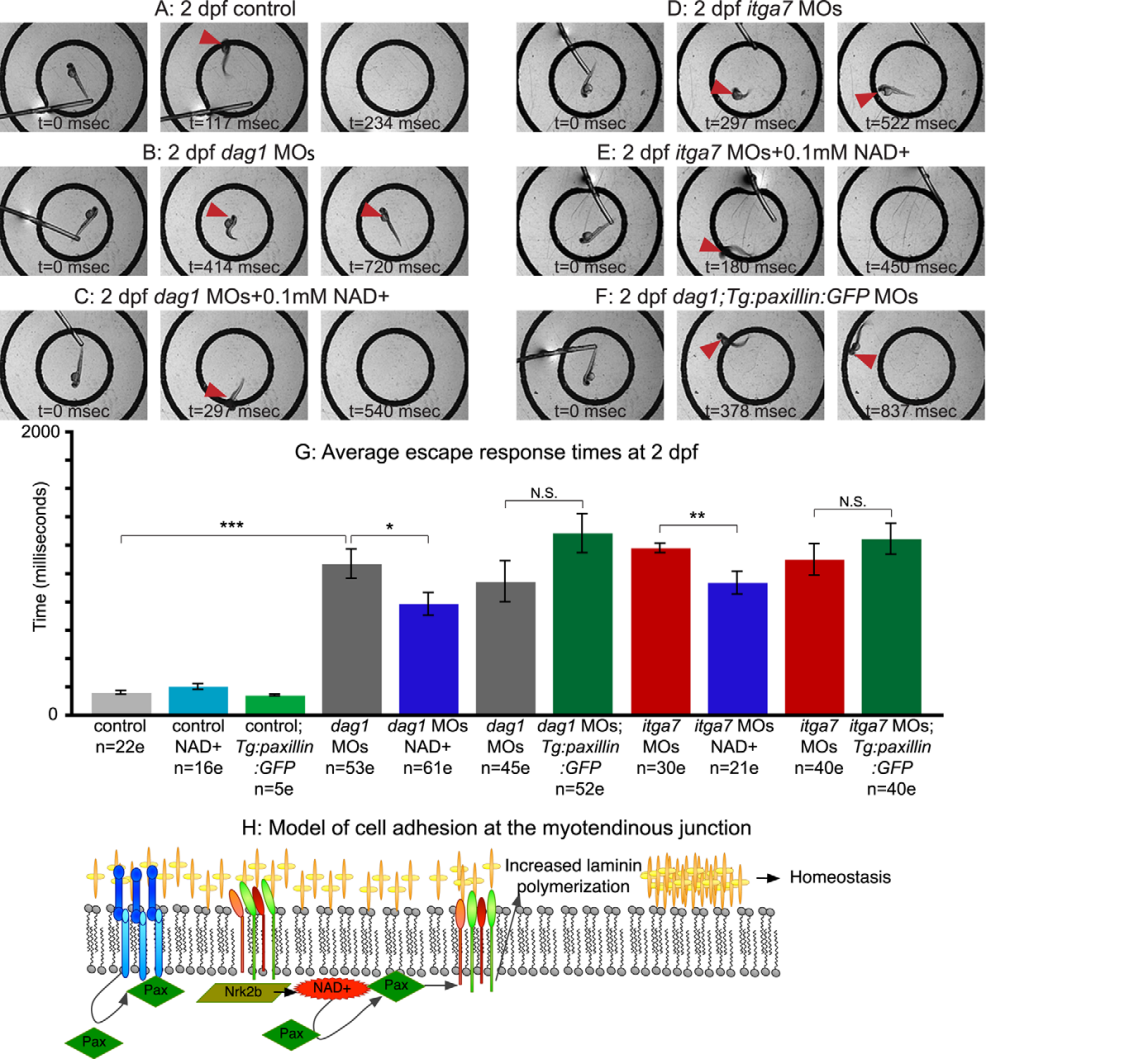
NAD+ supplementation, but not paxillin overexpression, improves motility of dystrophic zebrafish. (A–F) Individual panels from videos of escape responses after a touch stimulus at 2 dpf; time in milliseconds is denoted on panels. The outer circle is 10 mm in diameter. Red arrowheads point to the embryo's location. (A) Control embryo. (B) *dag1* morphant. (C) NAD+-supplemented *dag1* morphant. (D) *itga7* morphant. (E) NAD+-supplemented *itga7* morphant. (F) *dag1* MOs;*Tg:paxillin:GFP* embryo. (G) Average escape response times of 2 dpf dystrophic zebrafish after exogenous NAD+ treatment or overexpression of paxillin. Exogenous NAD+ or Emergen-C (not shown) significantly reduced the escape times of both *dag1* and *itga7* morphants. Overexpression of paxillin, however, did not reduce escape times of *dag1* or *itga7* morphants. *p<0.05; **p<0.01; ***p<0.001; N.S., not significant. (H) Model of cell adhesion at the MTJ. Our data show that laminin polymerization is necessary and sufficient for muscle fiber homeostasis and that NAD+ and paxillin increase laminin polymerization. We find that Dag1 and Nrk2b are required for paxillin localization to the MTJ. We hypothesize that NAD+, through mediating paxillin concentration at MTJs, invokes “inside-out” signaling through laminin receptors that results in increased laminin polymerization.

We next asked whether improvements in MTJ BM structure and maintenance of fiber attachment in dystrophic zebrafish correlated with physiological improvements by assaying the average time it takes for dystrophic embryos to swim a predetermined distance following a touch stimulus. Whereas control, NAD+-treated, Emergen-C-supplemented (unpublished data), or *Tg:Paxillin:GFP* control embryos always rapidly exited a 10 mm diameter circle (Figure 8G), only approximately half of *dag1* morphants swam out of the circle and were, on average, significantly slower than controls (Figure 8B,G; ***p<0.001). *Dag1* morphants supplemented with NAD+ (Figure 8C) or Emergen-C (unpublished data) were more likely to exit the circle compared to untreated *dag1* morphants and were, on average, significantly faster than untreated *dag1* morphants (Figure 8G; *p<0.05 for NAD+; **p<0.01 for Emergen-C). Similarly, NAD+-treated or Emergen-C-supplemented (unpublished data) *itga7* morphants were more likely to exit the circle (Figure 8E) and were, on average, significantly faster (Figure 8G; **p<0.01 for NAD+; *p<0.05 for Emergen-C) than unsupplemented *itga7* morphants (Figure 8D). These data suggest that NAD+ or Emergen-C treatment is sufficient to improve mobility in Dag1- or Itga7-deficient zebrafish. Interestingly, however, *Tg:paxillin:GFP* embryos injected with *dag1* MOs or *itga7* MOs actually tended to be slower than *dag1* or *itga7* morphants, respectively (Figure 8F,G). These results indicate that improving basement membrane structure in dystrophic zebrafish by overexpressing paxillin is not sufficient to improve mobility.

## Discussion

Tissue homeostasis, especially when tissue is stressed, requires cellular adaptations. Such adaptations are executed by a variety of mechanisms and lead to numerous potential outcomes such as hypertrophy, hyperplasia, and metaplasia, among others. Interactions between cells and their ECM microenvironment may play roles in skeletal muscle adaptation because cell-ECM adhesion complexes sense multiple types of physiological changes and interface with every major signaling pathway. Thus, cell-ECM adhesion complexes are in a prime position to facilitate appropriate responses to physiological change. However, in muscle disorders such as muscular dystrophies, sarcopenia, and denervation injuries, tissue structure is disrupted to such a degree that normal cellular adaptive responses are not sufficient to compensate. In zebrafish embryos deficient for Dag1 or Itga7, transmembrane receptor proteins involved in anchoring the sarcolemma of muscle cells to the BM, the consequence of disrupted cell adhesion is detachment and death of muscle fibers [30],[42]. Here, we show that addition of a small molecule, NAD+, is sufficient to increase the organization of the muscle cell microenvironment in dystrophic zebrafish. Exogenous NAD+ is sufficient to reduce dystrophy when either Itga7 or Dag1 is compromised because this pathway also functions through a different laminin receptor, Itga6. Pseudo-genetic epistasis experiments show that either Dag1 or Itga7 is required with Itga6 for the effects of exogenous NAD+ on reduction of dystrophy. Finally, we show that NAD+ plays a dual role in ameliorating dystrophy: NAD+-mediated paxillin clustering improves muscle structure and adhesion, but NAD+ acts independently of paxillin to improve motility. Taken together, these data provide fundamental new insights into the adaptability of cell adhesion mechanisms in vivo and the phenotypic consequences of this adaptability.

### The ECM Microenvironment: A Dynamic Structure Sufficient to Guide Morphological Change

A fundamental question is how tissue architecture is generated and maintained during development and homeostasis. The mechanical linkage of cells to BMs, substructures in the ECM, is necessary for cell viability and physiological homeostasis. Laminins are heterotrimeric proteins necessary for BM assembly. *laminin* mutations lead to multiple diseases including myopathies, junctional epidermolysis bullosa, laryngo-oncho-cutaneous syndrome, and microcoria-congenital nephritic syndrome [53]. Tissue-specific function of laminin isoforms arises not only through regulated expression but also through the generation of splice variants and post-translational glycosylation and processing. Despite the apparent tissue specificity of laminin function, multiple experiments indicate that, at least in the context of muscle biology, alternate BM components can partially compensate and restore cell-ECM adhesion when one laminin chain is disrupted. Mutations in *laminin alpha2* cause a severe congenital muscular dystrophy, MDC1A. Overexpression of either laminin alpha1, laminin-111, or mini-agrin, an ECM protein that can link with the DGC, slows the progression of dystrophy in *laminin alpha2* mutant mice [2],[3],[54],[55]. Recent data indicate that laminin-111 protein therapy can also reverse muscle disease even when the gene mutated encodes an intracellular protein. Injection of laminin-111 protein directly into muscle of DMD mice dramatically improves muscle structure and function by increasing Itga7 expression [56].

Here, we show that disorganized laminin-111 in the MTJ BM of Dag1-deficient zebrafish muscle tissue can be significantly improved by exogenous NAD+. We implicate laminin-111 organization in prolonging cell-ECM adhesion and fiber viability by showing that *laminin gamma1* is required for NAD+-mediated reduction of fiber detachment. Furthermore, we used genetic mosaic analysis to directly assess the role of a normally organized MTJ BM microenvironment on cell-ECM adhesion and muscle fiber viability in Dag1-deficient muscle cells. *dag1* MO-injected cells were transplanted into control embryos. Dag1-deficient muscle cells developed normally, and sarcomeres formed in the viable Dag1-deficient fibers. Significantly, only 1.9% of Dag1-deficient fibers in control embryos detached from the wild-type ECM microenvironment. Our findings suggest that proper organization of the ECM microenvironment plays a crucial role in maintenance of cell-ECM adhesion and fiber viability and thus muscle tissue homeostasis. Another striking example of how the ECM microenvironment impacts muscle cells is seen in how sarcomere morphogenesis changes depending upon the elastic moduli of hydrogels. The highest percent of sarcomerogenesis (60%) was observed after culture on a relatively elastic hydrogel (15 kPa) [57]. Taken together, these data suggest not only that the composition and physical properties of the ECM influence muscle structure but that local organization of the ECM can be dynamic and organizational state plays a critical role in muscle cell health. In addition, these data raise the possibility that a better understanding of the effects of ECM dynamics on cell and tissue morphogenesis and homeostasis, and the use of supplements like NAD+ to increase ECM organization, could improve in vitro tissue synthesis and regenerative medicine.

### Unique and Redundant Roles for the Laminin Receptor Proteins Integrin alpha7, Integrin alpha6, and Dystroglycan

Many studies have highlighted the complex, dynamic, and redundant interactions between integrin alpha7beta1 and the DGC in muscle disease. Although both of these laminin receptors have unique functions [18], they can partially compensate for each other in adhesion to laminin. Expression of the uncompromised complex's components can be upregulated in dystrophy, mutations in both complexes exacerbate dystrophy, and overexpression of alternate complex components can alleviate dystrophy [21]–[25]. Because exogenous NAD+ ameliorates dystrophy in embryos deficient for the DGC, we predicted that integrin alpha7beta1 would be required for reduction of dystrophy in *dag1* morphants. In support of this, we found that the frequency of myotomes with muscle degeneration in *dag1;itga7* double morphants was not significantly improved by NAD+. Surprisingly, however, overall muscle structure improved in NAD+-supplemented *dag1;itga7* double morphants. This result suggests (1) the existence of an additional laminin receptor downstream of NAD+ in the Nrk2b pathway, and (2) this receptor partially compensates when Dag1 and Itga7 are disrupted. We identified this receptor as Itga6. Compared to Itga7 and Dag1, far less is known about Itga6 function. Mutations in human *itga6* lead to epidermolysis bullosa with pyloric atresia (severe skin blistering with life-threatening obstruction of the digestive tract). The mouse *itga6* mutant is even more severe; mutant embryos die at birth with severe skin blistering [58]. Thus, at first glance, our results implicating Itga6 in muscle development and homeostasis are surprising. However, Itga6 is downstream of Myf5 and required for laminin assembly and normal myogenesis in mouse embryo explants [46]. Itga6 expression is a biomarker for highly myogenic cell populations in muscle tissue and required for normal myogenic differentiation and myotube formation of a porcine muscle progenitor cell population [47],[48]. Itga6 and laminin1 also play key roles in regeneration of CD117-positive cells in adult human pathological hearts [49]. These studies, along with our data, suggest that Itga6 does play a role in muscle physiology and may in fact be a key player in muscle tissue adaptation to stress.

Adhesion to laminin is required for initial MTJ development: MTJs in *laminin beta1* and *gamma1* mutant embryos are significantly wider than in wild-type embryos. The laminin receptors that mediate initial MTJ morphogenesis were not known. Our data indicate that Itga6 is the main laminin receptor mediating MTJ morphogenesis. MTJ angles are significantly wider in *itga6* morphants or *dag1;itga6* double morphant embryos compared to controls. MTJ development is not rescued when *itga6* or *dag1;itga6* morphants are supplemented with NAD+, indicating that Itga6 is necessary for the role of NAD+ in MTJ development. Although both *dag1* and *itga7* are expressed early in zebrafish development [30],[42], MTJ angles are normal in *dag1* or *itga7* single morphants. However, we show that MTJ angles are wider than normal in *dag1;itga7* double morphants. This result suggests that these two laminin receptors are required for normal MTJ development but function redundantly. MTJ angles in *dag1;itga7* double morphants are significantly narrower when incubated in NAD+, suggesting that Itga6 is sufficient to compensate for the decreased adhesion to laminin in *dag1;itga7* double morphants. Taken together, these results suggest that Itga6 is the major laminin receptor necessary and sufficient for mediating early muscle and MTJ morphogenesis, but that Itga7 and Dag1 also contribute.

Muscle homeostasis also requires adhesion to laminin. In this instance, however, integrin alpha7beta1 and the DGC are the main laminin receptors mediating muscle stability. Muscle degenerates in zebrafish embryos deficient for either *dag1* or *itga7* [30],[42]. Exogenous NAD+ increases laminin organization and reduces dystrophy in embryos deficient for either *dag1* or *itga7*. Pseudo-genetic epistasis analysis indicates that both *itga7* and *itga6* are necessary for NAD+-mediated amelioration of dystrophy in *dag1* morphants. Our data suggest that all three laminin receptors play unique roles in MTJ development and muscle homeostasis. However, when one receptor is compromised, the other receptors can partially compensate and NAD+ supplementation potentiates this compensatory response by facilitating increased laminin organization in an integrin-dependent manner.

### Paxillin and Basement Membrane Organization

Paxillin overexpression is also sufficient to restore laminin organization in dystrophic zebrafish. Paxillin is an essential signaling nexus that regulates cell adhesion, morphology, and migration [59]. Depending upon context, paxillin potentiates either the assembly or disassembly of cell-ECM adhesion complexes. The robust concentration of paxillin at MTJs during muscle development suggested a role for paxillin in maintaining muscle structure [60]. Here we show that overexpression of paxillin in *dag1* morphants increases organization of laminin-111 at the MTJ and reduces dystrophy. These results clearly implicate paxillin in playing a fundamental role in muscle homeostasis. It is very interesting that paxillin overexpression does not rescue morphogenesis/reduce dystrophy in *itga6* or *itga7* morphants. There are at least three possible explanations for why Itga6 and Itga7, but not Dag1, are required for paxillin overexpression-mediated restoration of muscle structure. One is that the subcellular localization of paxillin to MTJs is critical for paxillin function: paxillin localization is disrupted in *dag1* morphants, but not in *itga6* or *itga7* morphants. Overexpression of paxillin restores localization of paxillin to MTJs in *dag1* morphants and reduces dystrophy. Another scenario that would explain these results is that paxillin modulates “inside-out” signaling via Itga6 and Itga7. Paxillin localization to Itga6 and Itga7 containing cell-ECM adhesions may directly or indirectly cause these laminin receptors to adopt their high-affinity conformation, thus increasing adhesion to laminin. Finally, paxillin could be required to recruit the exocyst to the MTJ. The exocyst traffics vesicles to subdomains within the membrane and is critical for efficient exocytosis of integrins. Paxillin binding to an exocyst component, Sec5, mediates proper subcellular localization of exocyst complexes [61]. Thus, one mechanism by which paxillin could function upstream of Itga6 and Itga7 is modulation of exocyst localization. Intriguingly, another component of the exocyst is Arf6, which would require NAD+ as a cofactor for ADP-ribosylation of its targets. Our results clearly show that MTJ-localized paxillin is sufficient to impact local organization of the ECM microenvironment. It will be very interesting to determine the mechanism(s) of action and elucidate why paxillin—one of a thousand focal adhesion proteins—plays such a critical role. As paxillin is ubiquitously expressed and necessary for early development [62],[63], understanding how paxillin improves laminin organization in *dag1* morphants may provide insight into paxillin function in other tissues as well.

### Moving Toward Viable Therapeutic Options for Myopathies

Congenital muscular dystrophies are a heterogeneous group of early-onset progressive muscle-wasting diseases. In animal models of CMDs where sarcolemma integrity remains intact prior to fiber detachment, BM augmentation has been proposed and implemented as a successful approach to maintain muscle fiber viability. Interestingly, multiple different methods of BM augmentation have proved beneficial. In *laminin beta2* mutant zebrafish, initial failure between muscle fibers and the MTJ BM is compensated for by newly formed ectopic BMs at the detached ends of fibers [4]. Gene therapy approaches involving the expression of synthetic BM components are sufficient to reduce myopathy in mouse models [2],[3]. We show that modulation of the ECM microenvironment by the small molecule NAD+ also ameliorates muscle degeneration. In dystrophin-deficient zebrafish, where sarcolemma integrity is compromised prior to fiber detachment, a screen of chemicals approved for human use revealed that a nonselective phosphodiesterase inhibitor best restored muscle structure by activating cAMP-dependent PKA signaling [64]. These reports highlight the importance of classifying the etiology of fiber degeneration, as different myopathies may respond better to certain treatment approaches. In addition, investigation of combinatorial therapeutic approaches may prove most useful.

Along with muscle degeneration, CMDs can also present with joint and skeletal deformities and mental retardation. In dystrophic zebrafish, we find that NAD+ supplementation improves both muscle structure and mobility, but paxillin overexpression only improves muscle structure. Thus, in this system, structure does not beget function. It is perhaps not surprising that NAD+ contributes to the development and functioning of tissues other than muscle. Neurodegenerative disorders such as Parkinson's and Alzheimer's are associated with reduced NAD+ levels and increasing NAD+ metabolism slows neuronal degeneration in vitro. Although the exact mechanisms are not known, the neuroprotective effects of NAD+ are thought to be mediated by poly-ADP-ribose polymerases (PARPs), which play critical roles in genome stability, DNA repair, and telomere maintenance. In the case of ischemia, it is thought that the oxidative stress brought on by reperfusion with oxygen can cause DNA damage. This damage then activates the PARP-1 cascade and results in rapid depletion of NAD+. Energy within the cell quickly becomes limiting and may cause cell death. Evidence for this hypothesis comes from the fact that *PARP*−/− mice show reduced tissue damage and protected NAD+ metabolism in cerebral ischemia [65]. In a similar vein, nicotinamide treatment has been shown to allay the effects of fetal alcohol syndrome in mice [66]. This may be an important intervention for early neuronal damage during fetal development. Given these data, it is tempting to speculate that NAD+ may contribute to neuromuscular junction development or prolong/enhance signaling through neuromuscular junctions, thus contributing to improved motility in dystrophic zebrafish. As many NAD+ biosynthetic enzymes and precursors/metabolites are both required and cytoprotective in muscle and neural tissues, downstream targets of NAD+ signaling are promising potential therapies for multiple symptoms of muscle degeneration, including fiber atrophy, reduced motor function, and mental retardation.

The experiments shown here were undertaken because we had previously shown that Nrk2b-mediated NAD+ biosynthesis is necessary for laminin organization during myotendinous junction development. An alternate method to NAD+ supplementation in vivo is vitamin supplementation with a precursor to NAD+, such as niacin. Water-soluble Emergen-C packets that contain niacin were added to ERM and were sufficient to reduce muscle degeneration in *dag1* morphants. This result suggests the hypothesis that maternal and fetal nutrition may partly explain the variable progression and age of onset of some dystrophies. Clearly, vitamins other than niacin in the packets could contribute to the reduction of dystrophy. However, given that the reduction in muscle degeneration is similar in NAD+-supplemented, Emergen-C-supplemented, and paxillin-overexpressing *dag1* morphants, it seems unlikely that other vitamins are causing this effect.

Niacin has been used clinically for decades to manage dyslipidemia [67]. Although the mechanism of action is not well understood, it is known that niacin increases high-density lipoprotein (HDL) independently of NAD+ synthesis. An extended release form of niacin, Niaspan, has been used in combination with statins that lower LDL levels to treat cardiovascular disease [68]. However, NIH recently aborted a clinical trial combining niacin and statin therapy because the combination therapy did not decrease cardiovascular events in patients with heart and vascular disease and the risk of stroke was slightly increased (http://www.nih.gov/news/health/may2011/nhlbi-26.htm). Thus, it is not clear whether niacin will continue to be prescribed for cardiovascular disease. However, recent experiments in rats suggest other possible therapeutic uses for niacin. Niaspan is neuroprotective after stroke [69] and slows the progression of chronic kidney disease [70]. Our data suggest that the currently tractable approach of vitamin supplementation may warrant continued investigation.

## Summary

One of the advantages of using a “4D” system such as muscle development in vivo is the depth of dynamic range that cells exhibit in response to changes in cell signaling, cell adhesion, and the biophysics of the surrounding microenvironment. These processes allow cells to adapt to changing conditions during development, aging, injury response, and repair. We show that one such adaptive response—an increase in ECM organization mediated by the Nrk2b pathway—is sufficient to improve the resilience of muscle in dystrophic zebrafish. There are three laminin receptors expressed in muscle. The “canonical” receptors, Dag1 and Itga7, are highly expressed and, when mutated, lead to congenital muscular dystrophies. Here we implicate a “non-canonical” laminin receptor, Itga6, in the Nrk2b pathway. Recent findings from mouse, pig, and human cells, along with our data utilizing zebrafish embryos, suggest a conserved and previously unrecognized role for Itga6 in promoting muscle regeneration and homeostasis. Genetic mosaic analysis shows that the main effect of the Nrk2b pathway is an increase in structure of the ECM. A series of pseudo-genetic epistasis experiments with these three laminin receptors indicate that Dag1 or Itga7 is required along with Itga6 for NAD+ to reduce dystrophy. Taken together, these data suggest that two out of three receptors are required in order to sufficiently bind laminin and increase ECM organization. Our data highlight the contribution of the Nrk2b-NAD+-laminin-paxillin-Itga6/Itga7 to cellular adaptive responses and suggest that this pathway may have therapeutic potential.

## Materials and Methods

### Zebrafish Husbandry/Mutant/Transgenic Lines

Embryos were obtained from natural spawnings of zebrafish kept on a 16 hour light/8 hour dark cycle. Embryos were kept at 28.5°C in ERM and staged according to [71]. Embryos stably overexpressing paxillin fused to GFP were obtained from pairwise spawnings of carriers from the F1 generation of the *Tg:paxillin:GFP* line described in [27]. *wi390*/*laminin gamma1*−/− embryos [72] were obtained from natural spawnings of identified heterozygotes.

### Morpholino (MO) Injection

Stable antisense MO oligonucleotides were obtained from Gene Tools, LLC, and hydrated with sterile water to form 1 mM stocks. The *dag1* translation-blocking MO sequence has been published [30] and 12.5 ng was injected per embryo. A splice blocking *itga7* MO has been described [42] and 12.5 ng was injected per embryo. In our hands the phenotype was subtle and dystrophy varied between biological replicates. Two *itga6* translation-blocking MOs had the following sequences: MO1 5′-AGCTCCATTGCCTGAAATGAATG-3′ and MO2 5′-CTGTTGTATGAAAAATATAGCCCTT-3′. These two MOs were co-injected so that embryos received 9 ng MO1 and 8 ng MO2. The Gene Tools standard control MO sequence is 5′-CCTCTTACCTCAGTTACAAGGGATA-3′. 17 ng of control MO was injected as a control for *itga6* MO injections. The *p53* MO sequence is 5′-CCCTTGCGAACTTACATCAAATTCT-3′. *p53* MO was co-injected 1∶1 with *itga6* MOs. MOs were injected into the yolk of 1–2 cell stage embryos using a MPPI-2 pressure injector from ASI. For experiments where paxillin:GFP transgenic and wild-type zebrafish were injected with *dag1*, *itga7*, or *itga6* MOs, embryos from separate spawnings of each line were combined prior to injection and separated during imaging based on GFP fluorescence.

### NAD+ and Emergen-C Treatment

10 mg aliquots of Beta-NAD (Sigma, stored at −20°C) were dissolved to 15 mM stocks in ERM. Twenty-five embryos per 60 mm Petri dish were grown in 5 mL of ERM with a final concentration of either 0 or 0.1 mM NAD. Embryos were treated from shield stage (6 hpf) through live imaging/fixation, and the media were made fresh and changed every 24 h. A new aliquot was used for each change of the NAD-containing media. Emergen-C (original formula, registered trademark of Alacer Corporation) supplementation followed the above protocol. Emergen-C powder was dissolved in ERM such that the final concentration of niacin that embryos were exposed to was 6.77 micromolar. This concentration was chosen because it is roughly equivalent to what the concentration of niacin would be in an adult human bloodstream after consuming the contents of one Emergen-C packet (assuming 6 L of blood volume).

### Phalloidin Staining and Immunohistochemistry

Embryos were fixed using 4% paraformaldehyde for 4 h at room temperature or overnight at 4°C. Embryos were washed out of 4% paraformaldehyde at least three times with Phosphate Buffered Saline-0.1% Tween20 (PBS-0.1%Tw). Prior to phalloidin staining, embryos were permeabilized for 1.5 h at room temperature with PBS-2%Trition. Embryos were incubated in Alexa fluor 488 or 546 phalloidin (Molecular Probes) at a 1∶20 dilution overnight at 4°C. Antibody staining followed phalloidin staining or began with an incubation in block (5% w/v Bovine Serum Albumin in PBS-Tw) for at least 1 h at room temperature. Embryos were incubated in primary antibody overnight at 4°C, washed in block for 2 to 8 h at room temperature, and then incubated in secondary antibody overnight at 4°C. Embryos were washed in PBS-0.1%Tw prior to imaging. Primary antibodies were polyclonal anti-laminin-111 (Thermo Scientific, 1∶50 dilution in block) and monoclonal anti-paxillin (BD Biosciences, 1∶50 dilution in block). Secondary antibodies were GAM/GAR 488, 546, and 633 (Invitrogen, 1∶200 dilution in block).

### Genetic Mosaic Analysis

1–2 cell stage embryos were injected with *dag1* MOs and 10,000 MW dextrans (Molecular Probes). Morphant cells were removed at the sphere stage and transplanted into unlabeled control hosts. Hosts were grown to the appropriate stage, fixed, and stained with phalloidin.

### Westerns

Immunodetection of paxillin was conducted using preparations from 2 dpf embryos as previously described [27]. Protein concentration was quantified using a Nanodrop spectrophotometer, and sample concentrations were normalized prior to loading. Band intensities were quantified in NIH Image J (http://lukemiller.org/journal/2007/08/quantifying-western-blots-without.html).

### In Situ Hybridization

ISHs were done as previously described [27] with serial washes into and out of 2X SSC occurring before incubation in anti-digoxygenin antibody.

### Two-Dimensional Wavelet Transform Modulus Maxima (2DWTMM)

This rigorous and objective method of image quantification was applied as previously described in [27],[36].

### Motility Assay

Two concentric circles were drawn on an overhead projector transparency. The inner circle with a diameter of 5 mm and the outer circle with a diameter of 10 mm. A 60 mm Petri dish containing ERM was placed on top of the concentric circles. An embryo was placed in the Petri dish and aligned to the middle of the inner circle. A video was recorded (see Imaging section below) of each embryo being poked with the end of fishing wire, and the time that it took for the embryo to leave the field of view was documented. Embryos with abnormal escape responses were poked multiple times, and the most robust escape response was recorded and used for analysis. For comparison of average escape response time between groups, embryos that were unable to exit the field of view were assigned a time value equal to that of the slowest embryo within an experiment that could exit the field of view (the value was determined after biological replicates of the same experiment had been combined).

### Imaging

Images were collected and processed as described in [27]. Videos were acquired on a Zeiss Discovery.V12 microscope with a high-speed digital camera.

### Statistical Analyses

Error is reported as standard error of the mean, and significance was calculated using two-tailed, paired Student *t* tests, with *p*<0.05 being considered significant.

### Measuring MTJ Angles

Early MTJ morphogenesis was assayed through MTJ angle measurements as previously described in [27].

### Electron Microscopy

Transmission electron microscopy was performed by Dr. Bryan D. Crawford. Samples were fixed in Karnovsky's fixative (2% paraformaldehyde from 16% stock, 2.5% glutaraldehyde from 25% stock, 5% sucrose, and 0.1% CaCl2 in 0.1 M cacodylate buffer (from 0.2 M cacodylate stock at pH 7.2)). Embryos in Karnovsky's fixative were shipped to the University of Fredericton, New Brunswick, where they were post-fixed in oxmium, dehydrated, embedded, sectioned (70 nm), stained, and imaged.

## Abbreviations

2DWTMM: 2-dimensional wavelet transform modulus maxima
BM: basement membrane
cAMP: cyclic adenosine monophosphate
CMD: congenital muscular dystrophy
DGC: dystrophin-glycoprotein complex
Dag1: dystroglycan
Dpf: days post-fertilization
ECM: extracellular matrix
ERM: embryo rearing medium
GAM: goat anti-mouse
GAR: goat anti-rabbit
GFP: green fluorescent protein
HDL: high-density lipoprotein
Hpf: hours post-fertilization
ISH: in situ hybridization
Itga6: integrin alpha6
Itga7: integrin alpha7
LDL: low-density lipoprotein
MDC1A: merosin-deficient congenital muscular dystrophy, autosomal recessive
MO: morpholino oligonucleotide
MTJ: myotendinous junction
MTJ BM: myotendinous junction basement membrane
MW: molecular weight
NAD+: nicotinamide adenine dinucleotide
Nrk2b: nicotinamide riboside kinase 2b
PBS: phosphate buffered saline
PKA: protein kinase A
PARP: poly ADP ribose polymerase
SSC: saline-sodium citrate buffer
Tg: transgenic

## References

[1] Morgan JE, Zammit PS (2010) Direct effects of the pathogenic mutation on satellite cell function in muscular dystrophy. Exp Cell Res 316: 3100–3108. 20546725 10.1016/j.yexcr.2010.05.014

[2] Moll J, Barzaghi P, Lin S, Bezakova G, Lochmüller H, et al (2001) An agrin minigene rescues dystrophic symptoms in a mouse model for congenital muscular dystrophy. Nature 413: 302–307. 11565031 10.1038/35095054

[3] Bentzinger CF, Barzaghi P, Lin S, Ruegg MA (2005) Overexpression of mini-agrin in skeletal muscle increases muscle integrity and regenerative capacity in laminin-α2-deficient mice. FASEB J 19: 934–942. 15923403 10.1096/fj.04-3376com

[4] Jacoby AS, Busch-Nentwich E, Bryson-Richardson RJ, Hall TE, Berger J, et al (2009) The zebrafish dystrophic mutant softy maintains muscle fibre viability despite basement membrane rupture and muscle detachment. Development 136: 3367–3376. 19736328 10.1242/dev.034561 PMC2739150

[5] Ibraghimov-Beskrovnaya OO, Ervasti JMJ, Leveille CJC, Slaughter CAC, Sernett SWS, et al (1992) Primary structure of dystrophin-associated glycoproteins linking dystrophin to the extracellular matrix. Nature 355: 696–702. 1741056 10.1038/355696a0

[6] Gee SHS, Montanaro FF, Lindenbaum MHM, Carbonetto SS (1994) Dystroglycan-alpha, a dystrophin-associated glycoprotein, is a functional agrin receptor. Cell 77: 675–686. 8205617 10.1016/0092-8674(94)90052-3

[7] Yamada HH, Shimizu TT, Tanaka TT, Campbell KPK, Matsumura KK (1994) Dystroglycan is a binding protein of laminin and merosin in peripheral nerve. FEBS Lett 352: 49–53. 7925941 10.1016/0014-5793(94)00917-1

[8] Ervasti JM, Campbell KP (1993) A role for the dystrophin-glycoprotein complex as a transmembrane linker between laminin and actin. J Cell Biol 122: 809–823. 8349731 10.1083/jcb.122.4.809 PMC2119587

[9] Frost AR, Böhm SV, Sewduth RN, Josifova D, Ogilvie CM, et al (2010) Heterozygous deletion of a 2-Mb region including the dystroglycan gene in a patient with mild myopathy, facial hypotonia, oral-motor dyspraxia and white matter abnormalities. Eur J Hum Genet 18: 852–855. 20234391 10.1038/ejhg.2010.28 PMC2987357

[10] Hara Y, Balci-Hayta B, Yoshida-Moriguchi T, Kanagawa M, Beltrán-Valero de Bernabé D, et al (2011) A dystroglycan mutation associated with limb-girdle muscular dystrophy. N Engl J Med 364: 939–946. 21388311 10.1056/NEJMoa1006939 PMC3071687

[11] Inamori K-I, Yoshida-Moriguchi T, Hara Y, Anderson ME, Yu L, et al (2012) Dystroglycan function requires xylosyl- and glucuronyltransferase activities of LARGE. Science 335: 93–96. 22223806 10.1126/science.1214115 PMC3702376

[12] Godfrey C, Foley AR, Clement E, Muntoni F (2011) Dystroglycanopathies: coming into focus. Curr Opin Genet Dev 21: 278–285. 21397493 10.1016/j.gde.2011.02.001

[13] Mercuri E, Messina S, Bruno C, Mora M, Pegoraro E, et al (2009) Congenital muscular dystrophies with defective glycosylation of dystroglycan: a population study. Neurology 72: 1802–1809. 19299310 10.1212/01.wnl.0000346518.68110.60

[14] Jimenez-Mallebrera C, Torelli S, Feng L, Kim J, Godfrey C, et al (2009) A comparative study of alpha-dystroglycan glycosylation in dystroglycanopathies suggests that the hypoglycosylation of alpha-dystroglycan does not consistently correlate with clinical severity. Brain Pathol 19: 596–611. 18691338 10.1111/j.1750-3639.2008.00198.x PMC2860390

[15] Steffen LS, Guyon JR, Vogel ED, Beltre R, Pusack TJ, et al (2007) Zebrafish orthologs of human muscular dystrophy genes. BMC Genomics 8: 79. 17374169 10.1186/1471-2164-8-79 PMC1851013

[16] Moore CJ, Goh HT, Hewitt JE (2008) Genes required for functional glycosylation of dystroglycan are conserved in zebrafish. Genomics 92: 159–167. 18632251 10.1016/j.ygeno.2008.05.008

[17] Lin Y-Y, White RJ, Torelli S, Cirak S, Muntoni F, et al (2011) Zebrafish Fukutin family proteins link the unfolded protein response with dystroglycanopathies. Hum Mol Genet 20: 1763–1775. 21317159 10.1093/hmg/ddr059 PMC3071672

[18] Han R, Kanagawa M, Yoshida-Moriguchi T, Rader EP, Ng RA, et al (2009) Inaugural article: basal lamina strengthens cell membrane integrity via the laminin G domain-binding motif of α-dystroglycan. Proc Natl Acad Sci U S A 106: 12573–12579. 19633189 10.1073/pnas.0906545106 PMC2715328

[19] Mayer U, Saher G, Fässler R, Bornemann A, Echtermeyer F, et al (1997) Absence of integrin α7 causes a novel form of muscular dystrophy. Nat Genet 17: 318–323. 9354797 10.1038/ng1197-318

[20] Van Der Flier A, Gaspar A (1997) Spatial and temporal expression of the β1D integrin during mouse development. Dev Dyn 102: 472–486. 10.1002/(SICI)1097-0177(199712)210:4<472::AID-AJA10>3.0.CO;2-9 9415431

[21] Banks GB, Combs AC, Chamberlain JR, Chamberlain JS (2008) Molecular and cellular adaptations to chronic myotendinous strain injury in mdx mice expressing a truncated dystrophin. Hum Mol Genet 17: 3975–3986. 18799475 10.1093/hmg/ddn301 PMC2638580

[22] Burkin DJ, Wallace GQ, Milner DJ, Chaney EJ, Mulligan JA, et al (2005) Transgenic expression of α7β1 integrin maintains muscle integrity, increases regenerative capacity, promotes hypertrophy, and reduces cardiomyopathy in dystrophic mice. Am J Pathol 166: 253–263. 15632017 10.1016/s0002-9440(10)62249-3 PMC1602287

[23] Doe JA, Wuebbles RD, Allred ET, Rooney JE, Elorza M, et al (2011) Transgenic overexpression of the α7 integrin reduces muscle pathology and improves viability in the dyW mouse model of merosin-deficient congenital muscular dystrophy type 1A. J Cell Sci 124: 2287–2297. 21652631 10.1242/jcs.083311 PMC3113674

[24] Rooney JE, Welser JV, Dechert MA, Flintoff-Dye NL, Kaufman SJ, et al (2006) Severe muscular dystrophy in mice that lack dystrophin and alpha7 integrin. J Cell Sci 119: 2185–2195. 16684813 10.1242/jcs.02952

[25] Guo C (2006) Absence of α7 integrin in dystrophin-deficient mice causes a myopathy similar to Duchenne muscular dystrophy. Hum Mol Genet 15: 989–998. 16476707 10.1093/hmg/ddl018

[26] Snow CJ, Henry CA (2009) Dynamic formation of microenvironments at the myotendinous junction correlates with muscle fiber morphogenesis in zebrafish. Gene Expr Patterns 9: 37–42. 18783736 10.1016/j.gep.2008.08.003 PMC2655214

[27] Goody MF, Kelly MW, Lessard KN, Khalil A, Henry CA (2010) Nrk2b-mediated NAD+ production regulates cell adhesion and is required for muscle morphogenesis in vivo: Nrk2b and NAD+ in muscle morphogenesis. Dev Biol 344: 809–826. 20566368 10.1016/j.ydbio.2010.05.513 PMC2917104

[28] Bieganowski P, Brenner C (2004) Discoveries of nicotinamide riboside as a nutrient and conserved NRK genes establish a Preiss-Handler independent route to NAD+ in fungi and humans. Cell 117: 495–502. 15137942 10.1016/s0092-8674(04)00416-7

[29] Tempel W, Rabeh WM, Bogan KL, Belenky P, Wojcik M, et al (2007) Nicotinamide riboside kinase structures reveal new pathways to NAD. PloS Biol 5: e263 doi:10.1371/journal.pbio.0050263.sg001. 17914902 10.1371/journal.pbio.0050263 PMC1994991

[30] Parsons M, Campos I, Hirst E, Stemple D (2002) Removal of dystroglycan causes severe muscular dystrophy in zebrafish embryos. Development 129: 3505–3512. 12091319 10.1242/dev.129.14.3505

[31] Li J, Rao H, Burkin D, Kaufman SJ, Wu C (2003) The muscle integrin binding protein (MIBP) interacts with alpha7beta1 integrin and regulates cell adhesion and laminin matrix deposition. Dev Biol 261: 209–219. 12941630 10.1016/s0012-1606(03)00304-x

[32] Williamson R, Henry M, Daniels K (1997) Dystroglycan is essential for early embryonic development: disruption of Reichert's membrane in Dag1-null mice. Human Molecular Genetics 6: 831–841. 9175728 10.1093/hmg/6.6.831

[33] Sztal T, Berger S, Currie PD, Hall TE (2011) Characterization of the laminin gene family and evolution in zebrafish. Dev Dyn 240: 422–431. 21246659 10.1002/dvdy.22537

[34] Arnedo A, Decoster N, Roux S (2000) A wavelet-based method for multifractal image analysis. I. Methodology and test applications on isotropic and anisotropic random rough surfaces. Eur Phys J B 15: 567–600.

[35] Khalil A, Joncas G, Nekka F, Kestener P, Arneodo A (2006) Morphological analysis of H iFeatures. II. Wavelet-based multifractal formalism. Astrophys J Suppl S 165: 512–550.

[36] Snow CJ, Goody M, Kelly MW, Oster EC, Jones R, et al (2008) Time-lapse analysis and mathematical characterization elucidate novel mechanisms underlying muscle morphogenesis. PLoS Genet 4: e1000219 doi:10.1371/journal.pgen.1000219. 18833302 10.1371/journal.pgen.1000219 PMC2543113

[37] Hasegawa T, Matsumura K, Hashimoto T, Ikehira H, Fukuda H, et al (1992) Intramuscular degeneration process in Duchenne muscular dystrophy—investigation by longitudinal MR imaging of the skeletal muscles. Rinsho Shinkeigaku 32: 333–335. 1628461

[38] Nagao H, Morimoto T, Sano N (1991) Magnetic resonance imaging of skeletal muscle in patients with Duchenne muscular dystrophy—serial axial and sagittal section studies. Brain 23: 39–43. 1994993

[39] Hall TE, Bryson-Richardson RJ, Berger S, Jacoby AS, Cole NJ, et al (2007) The zebrafish candyfloss mutant implicates extracellular matrix adhesion failure in laminin alpha2-deficient congenital muscular dystrophy. Proc Natl Acad Sci U S A 104: 7092–7097. 17438294 10.1073/pnas.0700942104 PMC1855385

[40] Granato M, Van Eeden F, Schach U, Trowe T (1996) Genes controlling and mediating locomotion behavior of the zebrafish embryo and larva. Development 123: 399–413. 9007258 10.1242/dev.123.1.399

[41] Hayashi YK, Chou F-L, Engvall E, Ogawa M, Matsuda C, et al (1998) Mutations in the integrin α7 gene cause congenital myopathy. Nat Genet 19: 94–97. 9590299 10.1038/ng0598-94

[42] Postel R, Vakeel P, Topczewski J, Knöll R, Bakkers J (2008) Zebrafish integrin-linked kinase is required in skeletal muscles for strengthening the integrin-ECM adhesion complex. Dev Biol 318: 92–101. 18436206 10.1016/j.ydbio.2008.03.024

[43] Parsons MJ, Pollard SM, Saúde L, Feldman B, Coutinho P, et al (2002) Zebrafish mutants identify an essential role for laminins in notochord formation. Development 129: 3137–3146. 12070089 10.1242/dev.129.13.3137

[44] Humphries JD, Byron A, Humphries MJ (2006) Integrin ligands at a glance. J Cell Sci 119: 3901–3903. 16988024 10.1242/jcs.03098 PMC3380273

[45] Terpe HJ, Stark H, Ruiz P, Imhof BA (1994) Alpha 6 integrin distribution in human embryonic and adult tissues. Histochem Cell Biol 101: 41–49. 10.1007/BF00315830 8026982

[46] Bajanca F, Luz M, Raymond K, Martins GG, Sonnenberg A, et al (2006) Integrin alpha6beta1-laminin interactions regulate early myotome formation in the mouse embryo. Development 133: 1635–1644. 16554364 10.1242/dev.02336

[47] Wilschut KJ, Haagsman HP, Roelen BAJ (2010) Extracellular matrix components direct porcine muscle stem cell behavior. Exp Cell Res 316: 341–352. 19853598 10.1016/j.yexcr.2009.10.014

[48] Wilschut KJ, van Tol HTA, Arkesteijn GJA, Haagsman HP, Roelen BAJ (2011) Alpha 6 integrin is important for myogenic stem cell differentiation. Stem Cell Res 7: 112–123. 21763619 10.1016/j.scr.2011.05.001

[49] Castaldo C, Di Meglio F, Nurzynska D, Romano G, Maiello C, et al (2008) CD117-positive cells in adult human heart are localized in the subepicardium, and their activation is associated with laminin-1 and alpha6 integrin expression. Stem Cells 26: 1723–1731. 18436868 10.1634/stemcells.2007-0732

[50] Dolez M, Nicolas J-F, Hirsinger E (2011) Laminins, via heparan sulfate proteoglycans, participate in zebrafish myotome morphogenesis by modulating the pattern of Bmp responsiveness. Development 138: 97–106. 21115608 10.1242/dev.053975

[51] Eisen JS, Smith JC (2008) Controlling morpholino experiments: don't stop making antisense. Development 135: 1735–1743. 18403413 10.1242/dev.001115

[52] Robu ME, Larson JD, Nasevicius A, Beiraghi S, Brenner C, et al (2007) p53 activation by knockdown technologies. PLoS Genet 3: e78 doi:10.1371/journal.pgen.0030078. 17530925 10.1371/journal.pgen.0030078 PMC1877875

[53] Tzu J, Marinkovich MP (2008) Bridging structure with function: structural, regulatory, and developmental role of laminins. Int J Biochem Cell Biol 40: 199–214. 17855154 10.1016/j.biocel.2007.07.015 PMC2192629

[54] Gawlik K, Miyagoe-Suzuki Y, Ekblom P, Takeda S, Durbeej M (2004) Laminin α1 chain reduces muscular dystrophy in laminin α2 chain deficient mice. Hum Mol Genet 13: 1775–1784. 15213105 10.1093/hmg/ddh190

[55] Rooney JE, Knapp JR, Hodges BL, Wuebbles RD, Burkin DJ (2012) Laminin-111 protein therapy reduces muscle pathology and improves viability of a mouse model of merosin-deficient congenital muscular dystrophy. Am J Pathol 180: 1593–1602. 22322301 10.1016/j.ajpath.2011.12.019 PMC3349899

[56] Rooney JE, Gurpur PB, Burkin DJ (2009) Laminin-111 protein therapy prevents muscle disease in the mdx mouse model for Duchenne muscular dystrophy. Proc Natl Acad Sci U S A 106: 7991–7996. 19416897 10.1073/pnas.0811599106 PMC2683113

[57] Serena E, Zatti S, Reghelin E, Pasut A, Cimetta E, et al (2010) Soft substrates drive optimal differentiation of human healthy and dystrophic myotubes. Integr Biol (Camb) 2: 193–201. 20473399 10.1039/b921401a

[58] Georges-Labouesse E, Messaddeq N, Yehia G (1996) Absence of integrin α6 leads to epidermolysis bullosa and neonatal death in mice. Nat Genet 13: 370–373. 8673141 10.1038/ng0796-370

[59] Deakin NO, Turner CE (2008) Paxillin comes of age. J Cell Sci 121: 2435–2444. 18650496 10.1242/jcs.018044 PMC2522309

[60] Crawford BD, Henry CA, Clason TA, Becker AL, Hille MB (2003) Activity and distribution of paxillin, focal adhesion kinase, and cadherin indicate cooperative roles during zebrafish morphogenesis. Mol Biol Cell 14: 3065–3081. 12925747 10.1091/mbc.E02-08-0537 PMC181551

[61] Spiczka KS, Yeaman C (2008) Ral-regulated interaction between Sec5 and paxillin targets Exocyst to focal complexes during cell migration. J Cell Sci 121: 2880–2891. 18697830 10.1242/jcs.031641 PMC4445373

[62] Hagel M, George EL, Kim A, Tamimi R, Opitz SL, et al (2002) The adaptor protein paxillin is essential for normal development in the mouse and is a critical transducer of fibronectin signaling. Mol Cell Biol 22: 901–915. 11784865 10.1128/MCB.22.3.901-915.2002 PMC133539

[63] Mazaki Y (1998) Paxillin isoforms in mouse. Lack of the gamma isoform and developmentally specific beta isoform expression. J Biol Chem 273: 22435–22441. 9712867 10.1074/jbc.273.35.22435

[64] Kawahara G, Karpf JA, Myers JA, Alexander MS, Guyon JR, et al (2011) Drug screening in a zebrafish model of Duchenne muscular dystrophy. Proc Natl Acad Sci U S A 108: 5331–5336. 21402949 10.1073/pnas.1102116108 PMC3069215

[65] Eliasson M, Sampei K, Mandir A (1997) Poly (ADP-ribose) polymerase gene disruption renders mice resistant to cerebral ischemia. Nature 3: 1089–1095. 10.1038/nm1097-1089 9334719

[66] Ieraci A, Herrera DG (2006) Nicotinamide protects against ethanol-induced apoptotic neurodegeneration in the developing mouse brain. PLoS Med 3: e101 doi:10.1371/journal.pmed.0030101. 16478293 10.1371/journal.pmed.0030101 PMC1370925

[67] Altschul R, Hoffer A, Stephen JD (1955) Influence of nicotinic acid on serum cholesterol in man. Arch Biochem Biophys 54: 558–559. 14350806 10.1016/0003-9861(55)90070-9

[68] Hausenloy DJ, Yellon DM (2009) Enhancing cardiovascular disease risk reduction: raising high-density lipoprotein levels. Curr Opin Cardiol 24: 473–482. 19574922 10.1097/HCO.0b013e32832ebfe7

[69] Shehadah A, Chen J, Zacharek A, Cui Y, Ion M, et al (2010) Niaspan treatment induces neuroprotection after stroke. Neurobiol Dis 40: 277–283. 20554037 10.1016/j.nbd.2010.05.034 PMC2926170

[70] Cho K-H, Kim H-J, Kamanna VS, Vaziri ND (2010) Niacin improves renal lipid metabolism and slows progression in chronic kidney disease. Biochim Biophys Acta 1800: 6–15. 19878707 10.1016/j.bbagen.2009.10.009

[71] Kimmel CB, Ballard WW, Kimmel SR, Ullmann B, Schilling TF (2005) Stages of embryonic development of the zebrafish. Dev Dyn 203: 253–310. 10.1002/aja.1002030302 8589427

[72] Wiellette E, Grinblat Y, Austen M, Hirsinger E (2004) Combined haploid and insertional mutation screen in the zebrafish. Genesis 40: 231–240. 15593329 10.1002/gene.20090

